# The Epigenetic Aging–Cancer Continuum: Biomarkers, Metabolism, and Therapy

**DOI:** 10.3390/genes17091130

**Published:** 2026-09-16

**Authors:** Christos Papaneophytou, Myrtani Pieri, Maria-Eleni Makreli, Evelina Charidemou, Eleni P. Andreou

**Affiliations:** Department of Life Sciences, School of Life and Health Sciences, University of Nicosia, 2417 Nicosia, Cyprus; pieri.m@unic.ac.cy (M.P.); makreli.m1@live.unic.ac.cy (M.-E.M.); charidemou.e@unic.ac.cy (E.C.); andreou.el@unic.ac.cy (E.P.A.)

**Keywords:** epigenetic aging, cancer, circulating biomarkers, nutrition, metabolism, therapeutic response

## Abstract

Aging and cancer form a biological continuum influenced by epigenomic changes, metabolic dysfunction, inflammation, cellular senescence, and loss of tissue homeostasis. Age-related epigenetic alterations can promote cancer, which exploits plasticity for evolution, immune evasion, metastasis, and resistance. Nutrition and metabolism affect this process through one-carbon metabolism, methyl-donor availability, acetyl-CoA and NAD^+^ balance, redox status, microbiome metabolites, and chromatin enzyme activity. Circulating biomarkers such as cell-free DNA methylation, mutation-based ctDNA, fragmentomic features, and non-coding RNAs can detect tumor and host changes linked to aging, inflammation, nutrition, and treatment with minimal invasiveness. This review explores the epigenetic aging–cancer link, how nutrition and metabolism modify pathways, and the potential of circulating biomarkers for diagnosis, prognosis, prediction, and monitoring. The focus is on epigenetic plasticity, drug-tolerant states, resistance, epigenetic drugs, metabolic targeting, and nutritional interventions. New technologies, including single-cell and spatial epigenomics, long-read sequencing, and multimodal computational approaches, aid biomarker discovery and clinical use. Challenges include variability, misclassification, heterogeneity, confounding, reverse causality, overfitting, and limited validation. Clinical applications need standard workflows, representative cohorts, transparent models, and proof that biomarker-guided strategies improve outcomes.

## 1. Introduction

For decades, aging and cancer were often studied as distinct biological trajectories. Aging was primarily viewed as a progressive loss of systemic function and tissue homeostasis, whereas cancer was understood as a disease of aberrant proliferation, clonal expansion, and adaptive metabolic fitness [1]. Advances in biogerontology and oncology have increasingly indicated that these processes converge through overlapping molecular and cellular dysfunction [2]. Among the mechanisms linking aging and cancer, progressive epigenomic remodeling is particularly important [3]. The epigenome responds dynamically to developmental and mitotic history, cellular stress, inflammation, metabolic state, and environmental exposures, enabling adaptation but also creating permissive states for tissue dysfunction, malignant transformation, and therapeutic resistance [4].

Age-associated epigenetic remodeling includes loss of cellular identity, altered chromatin organization, and widespread changes in DNA methylation [5]. Epigenetic drift is characterized by global hypomethylation across repetitive and heterochromatic regions together with focal hypermethylation at selected CpG-rich loci, including developmental genes associated with Polycomb regulation [6,7]. DNA methylation-based epigenetic clocks further show that biological aging may diverge from chronological age. Epigenetic age acceleration (EAA) has been associated with age-related disease, multimorbidity, mortality, and, in some settings, cancer incidence or prognosis [8,9,10]. These associations support the value of epigenetic-aging measures as indicators of systemic biological vulnerability, although these associations do not establish EAA as a causal driver of malignant transformation [11].

Cancer can exploit the same loss of regulatory fidelity through non-mutational epigenetic reprogramming [12,13]. Altered DNA methylation, histone modifications, chromatin accessibility, and non-coding RNA (ncRNA) networks can suppress tumor-suppressive programs, activate oncogenic transcriptional states, facilitate immune evasion, sustain stem-like phenotypes, and promote therapeutic resistance [14,15]. These processes intersect with aging-associated inflammation, metabolic dysfunction, cellular senescence, and erosion of cellular identity [16]. The age-remodeled epigenome may therefore provide a permissive, although not independently sufficient, substrate for malignant transformation and tumor evolution, with the biological consequences determined by tissue type, genetic background, and environmental context [17].

This shared biology can also be interrogated through circulating biomarkers. Cell-free DNA (cfDNA) methylation, mutation-based circulating tumor DNA (ctDNA), cfDNA fragmentomics, circulating non-coding RNAs (c-ncRNAs), and extracellular vesicle (EV)-associated cargo provide complementary information on tissue of origin, tumor burden, clonal evolution, and treatment response [18,19]. These analytes are being investigated for early detection, prognosis, molecular residual disease (MRD) surveillance, recurrence monitoring, therapeutic stratification, and resistance assessment [20]. Their interpretation is complicated by the fact that circulating profiles also capture host aging, inflammation, tissue injury, metabolic dysfunction, and treatment exposure [21], making separation of tumor-derived and host-derived signals a central issue across the aging–cancer continuum.

Nutrition and metabolism provide a further mechanistic layer because chromatin-modifying enzymes depend on metabolites generated through one-carbon metabolism, intermediary metabolism, and redox regulation [22]. Folate, vitamin B12, choline, betaine, methionine, serine, and glycine contribute to methyl-group metabolism [23], whereas diet-derived bioactive compounds and microbiome-generated metabolites can influence histone acetylation, inflammatory signaling, immune function, and ncRNA regulation [24]. These pathways provide a biological basis through which nutritional and metabolic state may modify epigenetic aging, tumor behavior, and therapeutic response [25,26]. Their clinical implications, however, depend on biological context and the level of supporting evidence and are examined in detail in subsequent sections.

This review therefore examines the epigenetic aging–cancer continuum through three interconnected questions. First, it considers how aging-associated epigenetic remodeling may create vulnerabilities that are subsequently exploited during malignant transformation and how metabolic factors modify these processes. Second, it evaluates how tumor- and host-derived circulating signals can capture this biology and support cancer detection, monitoring, and therapeutic stratification. Third, it examines how epigenetic and metabolic plasticity contribute to drug tolerance and resistance and how these dependencies may be therapeutically targeted. Emerging single-cell, spatial, long-read, and multimodal approaches are considered in relation to the methodological and clinical requirements needed to translate these mechanisms into precision-oncology applications.

## 2. Shared Molecular Mechanisms of the Epigenetic Aging–Cancer Continuum: Drift, Plasticity, and Inflammaging

The transition from a healthy, homeostatic tissue state to malignancy is rarely driven by genetic alterations alone. It is also shaped by progressive changes in chromatin organization, DNA methylation, histone modifications, and ncRNA-mediated regulation [27]. Aging and cancer share a vulnerability stemming from reduced epigenetic fidelity: aging is associated with the gradual erosion of cellular identity and tissue resilience, whereas cancer exploits this altered regulatory landscape to support clonal expansion, phenotypic plasticity, immune evasion, and therapeutic resistance [28]. The aging epigenome may therefore provide a permissive, though not independently sufficient, substrate for malignant transformation and tumor evolution.

Importantly, mechanistic overlap between aging and cancer does not imply epigenetic equivalence. Shared or convergent alterations include heterochromatin disruption, repetitive-element derepression, global DNA hypomethylation, and focal hypermethylation at selected developmental and Polycomb-regulated loci, all of which can destabilize cellular identity and regulatory control [28,29,30,31]. By contrast, EAA and other clock-derived or age-associated methylation measures are primarily indicators of biological state and should not be interpreted as direct drivers of malignant transformation [8,9,10,11]. Some processes may also have opposing or stage-dependent consequences. Senescence, for example, initially restricts proliferation and can suppress tumor initiation, whereas persistence of senescent cells and their secretory phenotype may promote chronic inflammation, tissue remodeling, and tumor progression [32,33,34,35]. Similarly, Polycomb-mediated repression can preserve lineage identity in normal tissues but, when dysregulated in cancer, may reinforce stem-like or therapy-adaptive cell states; conversely, loss of Polycomb repressive complex 2 (PRC2) function can itself be tumor-promoting in specific molecular contexts [36]. The aging–cancer continuum should therefore be understood as convergence on selected epigenetic vulnerabilities whose biological consequences depend on cell type, tissue context, genetic background, and disease stage.

### 2.1. Epigenetic Drift and Heterochromatin Remodeling During Aging

Somatic cells accumulate progressive and partly stochastic alterations in epigenetic marks with age, a process commonly described as epigenetic drift. This remodeling is influenced by repeated cell division, imperfect maintenance of epigenetic information, chronic inflammation, oxidative stress, metabolic dysfunction, and environmental exposures. Rather than producing a uniform gain or loss of epigenetic information, aging redistributes DNA methylation and histone marks across the genome [37].

A prominent feature of the aging methylome is global hypomethylation, particularly across repetitive elements, intergenic regions, satellite DNA, and transposable elements such as long interspersed nuclear element-1 (LINE-1) [38]. These changes often accompany disruption of heterochromatin organization. In younger, homeostatic cells, DNA methylation and repressive chromatin marks keep repetitive genomic regions transcriptionally repressed, including constitutive heterochromatin marked by trimethylated histone H3 lysine 9 (H3K9me3) and facultative heterochromatin associated with Polycomb-mediated trimethylation of histone H3 lysine 27 (H3K27me3). With aging, weakening of these regulatory systems can reduce chromatin compaction, permit aberrant transcription from repetitive regions, and contribute to genomic instability [29].

Heterochromatin relaxation may also promote transposable-element derepression. In aging and senescent cells, LINE-1 transcription and the accumulation of cytoplasmic nucleic-acid intermediates can activate cyclic GMP–AMP synthase–stimulator of interferon genes (cGAS–STING) signaling and type I interferon responses. These events provide a mechanistic link among epigenetic drift, genome instability, sterile inflammation, and tissue aging [30].

Global hypomethylation is accompanied by focal hypermethylation at selected CpG-rich promoter regions [39]. These alterations frequently involve developmental and lineage-regulatory genes that are targets of Polycomb repression. PRC2, whose catalytic activity is mediated principally by enhancer of zeste homolog 2 (EZH2) or enhancer of zeste homolog 1 (EZH1), establishes H3K27me3 and thereby contributes to the maintenance of transcriptional programs and cellular identity. In stem and progenitor cells, many developmental promoters are maintained in a bivalent state characterized by the coexistence of repressive H3K27me3 and activating trimethylation of histone H3 lysine 4 (H3K4me3), allowing these genes to remain transcriptionally restrained yet responsive to differentiation signals [28,31].

Notably, age-associated DNA hypermethylation is preferentially enriched at Polycomb-targeted and bivalent promoters, particularly at developmental and lineage-specifying genes. Similar loci are also recurrently hypermethylated in cancer, suggesting a mechanistic intersection between epigenetic aging and malignant transformation. Progressive acquisition of DNA methylation at these normally plastic regulatory regions may convert relatively reversible Polycomb-mediated repression into more stable transcriptional silencing, thereby restricting differentiation potential and contributing to loss of cellular identity. Thus, Polycomb-regulated developmental loci represent a shared class of epigenetically vulnerable regions at which aging-associated drift and cancer-associated reprogramming may converge [28,31]. Age-associated focal hypermethylation of these loci therefore provides a plausible mechanistic bridge between progressive epigenetic remodeling and the distorted methylation landscape characteristic of many cancers.

### 2.2. Malignant Exploitation of Epigenetic Plasticity

Cancer cells exploit epigenetic plasticity to acquire adaptive phenotypes without requiring immediate changes in DNA sequence. Non-mutational reprogramming can alter transcriptional states, weaken differentiation barriers, and generate intratumoral heterogeneity, enabling malignant cells to adapt to hypoxia, nutrient limitation, immune surveillance, anticancer treatment, and metastatic colonization [40].

PRC2 provides a mechanistic example of how chromatin states altered during aging can be co-opted in cancer. Dysregulated PRC2/EZH1/EZH2 activity can reinforce H3K27me3-dependent repression of developmental and lineage-specifying programs, impair differentiation, maintain stem-like states, and facilitate adaptive cell-state transitions [36,41,42]. Increased EZH2 activity or expression occurs in several malignancies, whereas activating EZH2 mutations define subsets of lymphoid cancers [36,43]. Conversely, loss of PRC2 function can promote tumorigenesis in particular genetic contexts [36]. Thus, the consequences of Polycomb dysregulation are lineage- and context-dependent but illustrate how altered H3K27me3 control can contribute to loss of cellular identity and malignant phenotypic plasticity [36,42].

One of the best-characterized epigenetic alterations in cancer is focal promoter CpG-island hypermethylation, which can repress tumor-suppressor and DNA-repair genes. Context-specific examples include methylation-associated silencing of CDKN2A, MLH1, and BRCA1 [44]. The CpG island methylator phenotype (CIMP) was initially and most extensively characterized in colorectal cancer, where a CIMP-high subgroup is associated with BRAF V600E, MLH1 promoter methylation, and microsatellite instability [45]. A mechanistically related glioma CpG island methylator phenotype (G-CIMP) is strongly associated with IDH1 mutation and widespread DNA hypermethylation [46]. Concordant promoter-hypermethylation subgroups have also been described in gastric and endometrial adenocarcinomas and across multiple tumor types in pan-cancer analyses [47].

Cancer-associated DNA hypomethylation contributes to tumor progression by destabilizing repetitive regions, promoting chromosomal instability, and enabling aberrant transcriptional programs. Together, focal hypermethylation and global hypomethylation create an epigenetic landscape in which tumor-suppressive pathways may be silenced while normally repressed genomic regions become transcriptionally active [48].

Epigenetic plasticity also facilitates the acquisition and maintenance of cancer stem-like states. Normal differentiation is stabilized by epigenetic barriers that constrain cellular identity. During tumor progression, alterations in DNA methylation, Polycomb-mediated repression, histone modifications, chromatin accessibility, and ncRNA regulation can weaken or reconfigure these barriers and permit transitions among differentiated, proliferative, quiescent, invasive, and stem-like states. Such state switching contributes to intratumoral heterogeneity, metastatic competence, immune escape, and therapeutic resistance [42].

### 2.3. Cellular Senescence, SASP, and Inflammaging as a Common Soil

Cellular senescence provides an important biological bridge between epigenetic aging and cancer. Initially, senescence acts as a tumor-suppressive response by imposing stable cell-cycle arrest following oncogenic stress, DNA damage, telomere dysfunction, and other forms of cellular injury. This arrest is reinforced by epigenetic remodeling, including altered chromatin compaction and, in some contexts, the formation of senescence-associated heterochromatin foci that repress proliferation-related gene programs [32].

The long-term accumulation of senescent cells in aging tissues can nevertheless generate a microenvironment that supports malignant progression [33]. Senescent cells secrete cytokines, chemokines, growth factors, extracellular-matrix-remodeling enzymes, and inflammatory mediators collectively known as the senescence-associated secretory phenotype (SASP). Representative components include interleukin-6 (IL-6), IL-1β, IL-8, tumor necrosis factor alpha (TNF-α), and matrix metalloproteinases. Although transient SASP activity can contribute to wound repair and immune-mediated clearance of damaged cells, persistent signaling promotes chronic, sterile, low-grade inflammation, commonly termed inflammaging [34].

SASP factors can modify neighboring cells through paracrine signaling. Chronic exposure may activate nuclear factor kappa B (NF-κB), signal transducer and activator of transcription 3 (STAT3), and related inflammatory pathways, thereby altering transcriptional programs, chromatin states, immune interactions, and epithelial-cell behavior. In premalignant or genetically altered cells, these signals may facilitate clonal expansion, survival, epithelial–mesenchymal plasticity, and therapeutic resistance [49].

Inflammaging is also reinforced by epigenetic dysregulation [50]. Loss of repression at repetitive elements, including LINE-1, can activate innate immune pathways and amplify inflammatory signaling [51]. Epigenetic drift, heterochromatin remodeling, transposable-element activation, senescence, and SASP therefore form an interconnected network through which aging tissues may become increasingly permissive to cancer initiation and progression [16]. Because chromatin maintenance, senescence, inflammatory signaling, and cellular metabolism are interdependent, this network also provides a mechanistic link to the metabolic regulation discussed in Section 4.

Senescence therefore exemplifies the context-dependent nature of the aging–cancer continuum: a stress-responsive program that initially constrains malignant transformation can, when senescent cells persist, promote tumor evolution through chronic SASP signaling and remodeling of the surrounding tissue.

## 3. Circulating Biomarkers Across the Epigenetic Aging–Cancer Continuum

Translating cancer epigenetics clinically requires minimally invasive approaches that capture molecular changes across anatomical sites and over time. Although tissue biopsy remains essential for histopathological and molecular characterization, its spatial and temporal limitations can restrict assessment of tumor heterogeneity and treatment-associated evolution [52,53,54]. Liquid biopsy provides a complementary approach by analyzing tumor- and host-derived material released into blood and other body fluids, including cfDNA, ctDNA, extracellular RNAs, EVs, proteins, metabolites, and circulating cells [55]. The biological origin of circulating signals is central to their interpretation within the aging–cancer continuum. Mutation-based ctDNA is predominantly tumor-derived when variants are correctly assigned, whereas cfDNA methylation, fragmentomic features, and c-ncRNAs generally represent mixtures of malignant and non-malignant sources; blood-cell DNA methylation clocks primarily reflect host biological aging. Consequently, host age, immune-cell composition, inflammation, tissue injury, metabolic and nutritional state, medications, and treatment exposure can influence circulating measurements independently of tumor burden [56]. These analytes therefore provide distinct but potentially complementary information on the evolving tumor and host biological state. Table 1 summarizes their biological sources, susceptibility to host-related influences, principal strengths and limitations, and potential clinical applications.

Section 6 considers multimodal integration with clinical, pathological, imaging, metabolic, and nutritional information, while Section 7 addresses the requirements for translational validation.

### 3.1. Cell-Free DNA Methylation and Tissue-of-Origin Inference

Plasma cfDNA consists predominantly of fragments released through cell death, with hematopoietic cells contributing most of the background signal in healthy individuals and a variable tumor-derived fraction in patients with cancer [70]. ctDNA abundance varies with tumor type, anatomical site, stage, burden, cellular turnover, and treatment exposure and may be particularly low in localized or low-shedding disease [71]. Mutation-based assays provide high molecular specificity and can identify actionable or resistance-associated variants, but sensitivity may be limited by low tumor fraction, tumor heterogeneity, and the availability of trackable variants. Age-associated clonal hematopoiesis further complicates interpretation because some plasma variants may originate from expanded hematopoietic clones rather than the tumor [72].

cfDNA methylation provides complementary information by interrogating coordinated cancer-associated changes across numerous CpG sites and genomic regions, including focal regulatory hypermethylation and broader hypomethylation [73]. Because methylation patterns are closely linked to cellular identity, reference methylome atlases and computational deconvolution can estimate the tissues and cell populations contributing to the cfDNA pool [59,74]. This property supports tissue-of-origin inference after detection of a cancer-associated signal and may aid evaluation of cancers of unknown primary origin or multicancer early-detection results [75]. Assay performance nevertheless depends on locus selection, DNA input, tumor fraction, analytical chemistry, sequencing depth, and classifier generalizability [76].

Targeted methylation sequencing has demonstrated the feasibility of detecting cancer-associated signals across multiple tumor types while estimating their likely anatomical origin [57]. Such approaches may complement established organ-specific screening strategies [77], but performance observed in case–control or clinically enriched cohorts may not translate directly to population screening. Prospective studies must establish appropriate diagnostic pathways after a positive result and determine effects on stage distribution, overdiagnosis, cost-effectiveness, and cancer-related mortality [78].

Host aging further modifies the background against which tumor-associated methylation signals are detected. Age-related changes in blood-cell composition, methylation, inflammation, tissue turnover, metabolic disease, organ dysfunction, and treatment exposure can alter non-malignant contributions to plasma cfDNA and thereby complicate tumor-specific interpretation. Integration of methylation with mutation profiles, fragmentomic features, copy-number alterations, and other biomarkers may improve discrimination between tumor- and host-derived signals, particularly at low tumor fractions [79]; broader computational and clinical-validation requirements are considered in Section 6 and Section 7.

### 3.2. Blood-Cell DNA Methylation Clocks and Host Biological Aging

Cancer-associated cfDNA methylation signatures should be distinguished from epigenetic clocks. Chronological age denotes elapsed time since birth, whereas biological age reflects interindividual differences in physiological decline; epigenetic clocks provide one molecular estimate of this broader construct rather than a direct measure of biological age itself. First-generation clocks, including the Horvath and Hannum models, primarily predict chronological age from DNA methylation profiles. Second-generation measures, including PhenoAge and GrimAge, incorporate methylation surrogates associated with physiological dysregulation, morbidity, or mortality, whereas third-generation approaches estimate the pace of biological aging [80,81]. These generations therefore capture partly different biological features and may have distinct applications in oncology [80,81,82].

EAA has been associated in observational cohorts with mortality, multimorbidity, frailty, cardiovascular disease, and several cancers [83], but cancer-related associations vary with clock selection, cancer type, population, tissue source, blood-cell composition, treatment history, smoking, metabolic health, and other confounders [84]. First-generation clocks may be most informative for age-discordance analyses, whereas later-generation measures may better capture host vulnerability or mortality-associated risk. None is currently validated for individual cancer-risk prediction or as a surrogate endpoint for cancer outcomes; EAA should therefore be considered an investigational marker of systemic vulnerability rather than a clinical cancer-risk test [85,86].

The distinction between blood-cell DNA and plasma cfDNA is also critical. Leukocyte methylation measurements are strongly influenced by immune-cell composition and hematopoietic aging [87,88], whereas plasma cfDNA derives from multiple tissues, although hematopoietic cells remain the predominant source under many physiological conditions [70]. Blood-cell clocks and tumor-associated cfDNA methylation assays therefore measure related but biologically distinct processes. Smoking, inflammation, adiposity, metabolic dysfunction, and anticancer treatment can further alter blood-based clock estimates through both within-cell methylation changes and shifts in leukocyte composition.

Treatment-associated aging should likewise be distinguished from chronological age and baseline EAA. Cytotoxic therapy, radiotherapy, transplantation-related stress, and other exposures may alter methylation-based aging measures, but increases or decreases in clock-derived estimates do not necessarily indicate accelerated biological deterioration or rejuvenation. Such changes may instead reflect altered hematopoietic composition, inflammation, tissue injury, or recovery and should be interpreted alongside clinical and physiological outcomes.

Age-prediction models have also begun to be derived directly from plasma cfDNA methylation profiles, potentially capturing systemic aging, tissue injury, and disease-associated cell death within the same sample [89]. These approaches remain preliminary and require validation in large, longitudinal, and demographically diverse populations before incorporation into cancer-detection or treatment-monitoring platforms [90].

Within the aging–cancer continuum, blood-cell clocks are therefore most appropriately viewed as measures of host biological state that may complement tumor-specific biomarkers. Integrating cfDNA methylation with inflammatory and immune measures, body composition, nutritional and metabolic status, and treatment exposure may help characterize host vulnerability, treatment tolerance, or recovery but should not obscure the distinction between systemic aging and tumor-specific molecular signals.

### 3.3. Circulating Non-Coding RNAs and Extracellular Vesicles

Whereas DNA methylation provides relatively stable information on cellular identity and regulatory history, c-ncRNAs can reflect more dynamic changes in transcription, cellular stress, inflammation, metabolism, intercellular communication, and treatment response. The principal classes investigated as circulating cancer biomarkers include miRNAs, lncRNAs, and circRNAs [66]. Despite abundant plasma ribonuclease activity, selected ncRNAs remain detectable through association with EVs, RNA-binding proteins such as Argonaute 2, and lipoprotein particles [91,92]. EV is used here as the generic term unless endosomal biogenesis has been sufficiently demonstrated to classify a vesicle specifically as an exosome [93].

c-ncRNAs can originate from viable-cell secretion or from apoptosis, necrosis, inflammation, platelet activation, and tissue injury; altered plasma abundance therefore does not necessarily indicate tumor-specific release [94]. Hematopoietic cells, platelets, erythrocytes, endothelial cells, and metabolically active tissues can all contribute to circulating profiles, with hemolysis representing a particularly important source of variation for several miRNAs [95].

In cancer, ncRNAs have been associated with oncogenic signaling, hypoxia, angiogenesis, immune evasion, epithelial–mesenchymal plasticity, metastasis, and therapeutic resistance [66,96,97]. They are also influenced by aging and cellular senescence: age-associated changes have been reported for inflammatory and senescence-related miRNAs, including miR-21, miR-34a, miR-126, and miR-146a [98,99]. However, these signals vary with immune and cardiometabolic status, medications, physical activity, diet, and analytical conditions and should not be interpreted as universal markers of either biological aging or cancer [100].

Nutritional and metabolic state may further modify c-ncRNA profiles through inflammatory signaling, insulin sensitivity, lipid and redox metabolism, microbiome-derived metabolites, and tissue-specific transcription. Consequently, the same circulating RNA signal may reflect tumor activity, host metabolic state, or both, reinforcing the need to distinguish tumor-derived from host-derived biology across the aging–cancer continuum. Section 4 further discusses these metabolic interactions.

Senescent and malignant cells may also alter RNA cargo carried by EVs and other extracellular particles. Following uptake by recipient cells, these RNAs can influence inflammatory signaling, DNA-damage responses, stromal and immune-cell behavior, angiogenesis, and treatment sensitivity, providing a potential mechanistic link between senescence-associated inflammation and tumor-microenvironment remodeling [101]. Importantly, altered circulating abundance alone does not demonstrate functional intercellular transfer; mechanistic attribution requires evidence of uptake, intracellular localization, and functional effects in recipient cells.

### 3.4. Clinical Applications Across the Cancer Continuum

Circulating biomarkers are being investigated across risk assessment, early detection, prognosis, treatment monitoring, MRD surveillance, recurrence detection, and resistance assessment. Their clinical value depends on the biological information captured by each analyte and the decision it is intended to improve; molecular association or analytical detectability alone is insufficient without incremental information beyond established predictors or diagnostic pathways.

For risk assessment, blood-cell DNA methylation clocks may characterize population-level relationships between biological aging and cancer susceptibility, but their performance varies across clocks, cancer types, and populations and is insufficient for individual cancer screening [102,103]. cfDNA methylation is more directly relevant to early detection because cancer-associated methylation patterns can be interrogated across multiple genomic regions and may simultaneously provide tissue-of-origin information, including when tumor-derived DNA is scarce [104]. However, stage-dependent sensitivity, false-positive results, diagnostic follow-up, overdiagnosis, and host-related methylation variation remain important concerns. Methylation-based multicancer early-detection assays therefore require validation in populations representative of their intended clinical use [105].

Longitudinal circulating-biomarker measurements may also provide information on prognosis and treatment response. Mutation-based ctDNA and tumor-associated methylation signals can track molecular tumor burden more specifically than total cfDNA concentration, which may also increase during inflammation, infection, exercise, or tissue injury [106]. Declining tumor-associated signals during therapy may indicate molecular response, whereas persistence or re-emergence may indicate incomplete clearance, progression, or resistance [107]. c-ncRNA profiles can also change during chemotherapy, radiotherapy, immunotherapy, and targeted treatment [108], although host inflammation, metabolic change, tissue injury, and treatment toxicity complicate tumor-specific interpretation. Serial measurements are therefore generally more informative than isolated values, but their use to guide treatment modification requires context-specific clinical validation.

MRD represents one of the most clinically advanced applications of serial liquid biopsy. Tumor-informed and tumor-naïve approaches using somatic variants, methylation signatures, fragmentomic features, or combinations thereof are being evaluated after definitive treatment [109]. In a prospective longitudinal study of stage I–III colorectal cancer, postoperative positivity using a six-marker ctDNA methylation assay was strongly associated with recurrence, while longitudinal testing detected recurrence a median of approximately three months before radiological confirmation [58,110]. Nevertheless, sensitivity depends on residual tumor burden, anatomical site, sampling time, plasma volume, and tumor shedding, and a negative result does not exclude residual disease. Although postoperative ctDNA positivity consistently identifies patients at increased recurrence risk, whether MRD-guided treatment escalation or de-escalation improves survival remains context-dependent and not yet fully established [111].

Circulating biomarkers may additionally support resistance monitoring and precision oncology. Mutation-based ctDNA can identify actionable alterations and emerging resistance-associated clones, whereas methylation, fragmentomic, and c-ncRNA profiles may capture non-mutational adaptation, including lineage plasticity, stem-like reprogramming, immune evasion, and metabolic change [112,113]. Integrating genomic and epigenetic signals may therefore help distinguish stable clonal selection from reversible phenotypic adaptation, although prospective evidence is required before such approaches can routinely guide treatment selection or modification [114]. Section 5 discusses the underlying mechanisms of therapeutic plasticity.

Across these applications, tumor-derived signals must be distinguished from host-related variation. cfDNA assays are influenced by tumor fraction, clonal hematopoiesis, sequencing depth, and assay-specific effects, whereas c-ncRNA and EV measurements are particularly sensitive to hemolysis, platelet contamination, carrier heterogeneity, extraction, and normalization [20,66,115,116]. Host age, inflammation, metabolic status, organ function, medications, and treatment exposure can further alter circulating profiles independently of tumor burden [117,118]. Section 7 addresses these cross-cutting requirements for analytical validity, confounding control, reproducibility, and clinical utility. Thus, the greatest clinical potential of circulating biomarkers lies not in any single analyte, but in combining biologically distinct tumor- and host-derived signals within clearly defined clinical contexts.

## 4. Nutrition and Metabolism as Modifiers of the Epigenetic Aging–Cancer Continuum

Epigenomic remodeling during aging and cancer does not occur independently of cellular metabolism. Chromatin-regulating enzymes operate within a metabolically responsive environment and depend on substrates, cofactors, and inhibitors generated through intermediary metabolism [119]. DNA methyltransferases (DNMTs) use S-adenosylmethionine (SAM) as a methyl donor; histone acetyltransferases (HATs) transfer acetyl groups from acetyl-CoA; sirtuin deacetylases require nicotinamide adenine dinucleotide (NAD^+^); ten–eleven translocation (TET) enzymes and Jumonji C domain-containing demethylases require Fe^2+^, molecular oxygen, and α-ketoglutarate; and lysine-specific demethylase 1 (LSD1) uses flavin adenine dinucleotide (FAD) as a cofactor [120,121]. Changes in nutrient availability, mitochondrial function, redox state, and metabolic flux can therefore influence epigenetic regulation by altering the production, distribution, and local availability of these metabolites. This relationship is reciprocal: epigenetic reprogramming can in turn alter the expression of genes controlling glycolysis, mitochondrial function, lipid metabolism, one-carbon metabolism, and redox homeostasis, thereby reshaping the metabolic state of aging and malignant cells [122].

This metabolic sensitivity provides an important connection among aging, nutrition, and cancer. Aging can alter dietary intake, nutrient absorption, mitochondrial function, organ reserve, inflammatory state, and metabolite handling. Cancer further reprograms nutrient utilization to sustain proliferation, redox balance, biomass production, and adaptation to treatment. Nutritional deficiency or excess, metabolic disease, and microbiome-derived metabolites may consequently influence DNA methylation, histone modifications, chromatin accessibility, and ncRNA expression within both host and tumor compartments [123]. Conversely, tumor-associated epigenetic states can reinforce metabolic programs that support survival under nutrient limitation, oxidative stress, and therapeutic pressure, making epigenetic–metabolic plasticity a bidirectional component of the aging–cancer continuum.

These relationships are highly context-dependent. Dietary intake does not translate directly into nuclear metabolite concentrations, and the effect of a nutrient depends on tissue type, genotype, age, baseline nutritional status, microbiome composition, organ function, disease stage, medication use, and tumor metabolism. Nutrition should therefore be regarded as a potentially modifiable influence on the epigenetic aging–cancer continuum rather than a deterministic controller of biological aging, cancer risk, or therapeutic response [124].

### 4.1. Metabolite-Dependent Regulation of the Epigenome

The epigenome acts partly as a biochemical sensor of cellular metabolic state because many chromatin-modifying enzymes depend directly on metabolites generated through glycolysis, the tricarboxylic acid (TCA) cycle, amino-acid metabolism, and mitochondrial redox reactions [125]. These metabolites, however, do not function as simple systemic switches: their epigenetic effects depend on intracellular compartmentalization, metabolic flux, local enzyme recruitment, competing pathways, and nuclear metabolite availability.

SAM is the principal methyl donor used by DNA, RNA, protein, and histone methyltransferases. The product of these reactions, S-adenosylhomocysteine (SAH), inhibits methyltransferase activity. The SAM:SAH ratio is therefore commonly used as an indicator of cellular methylation potential and provides a direct biochemical link between one-carbon metabolism and epigenetic regulation [126]. Its nutritional and cancer-related implications are considered in Section 4.2.

Acetyl-CoA links carbon metabolism to chromatin accessibility. Cytosolic and nuclear acetyl-CoA can be generated through ATP-citrate lyase, acetyl-CoA synthetases, and related pathways. Increased local availability can support histone acetylation and transcriptionally permissive chromatin, whereas nutrient deprivation or metabolic stress may reduce acetyl-CoA availability and alter acetylation patterns. These effects can be spatially and locus-specific because metabolic enzymes may localize to chromatin and generate metabolites in proximity to chromatin-modifying complexes [127].

NAD^+^ provides another connection between nutrient sensing, mitochondrial function, and chromatin regulation. Sirtuins consume NAD^+^ during deacetylation and thereby respond to the cellular redox state and metabolic activity. Age-associated disruption of NAD^+^ metabolism may influence DNA repair, mitochondrial function, inflammatory signaling, and chromatin stability [128]. However, modulation of NAD^+^ metabolism is tissue- and disease-context dependent, and supplementation with NAD^+^ precursors cannot be assumed to produce uniform epigenetic or clinical effects, particularly in individuals with established cancer or elevated cancer susceptibility [129].

TET enzymes and Jumonji C domain-containing histone demethylases require α-ketoglutarate and can be inhibited by structurally related metabolites, including succinate, fumarate, and 2-hydroxyglutarate [130,131]. Accumulation of these metabolites in tumors with isocitrate dehydrogenase mutations or loss of fumarate hydratase or succinate dehydrogenase activity can disrupt DNA and histone demethylation and produce extensive epigenetic reprogramming [132]. These tumor-intrinsic examples provide direct evidence that altered metabolic flux can reprogram the epigenome independently of dietary exposure.

Taken together, metabolite-sensitive chromatin regulation links one-carbon metabolism, acetyl-CoA availability, NAD^+^-dependent deacetylation, and TCA-cycle-derived metabolites to the epigenetic state. The reciprocal direction, how epigenetic programs reshape glycolysis, mitochondrial function, lipid metabolism, redox homeostasis, and treatment adaptation, is developed in the following subsections.

### 4.2. One-Carbon Metabolism and Methyl-Donor Balance

**Figure 1:**
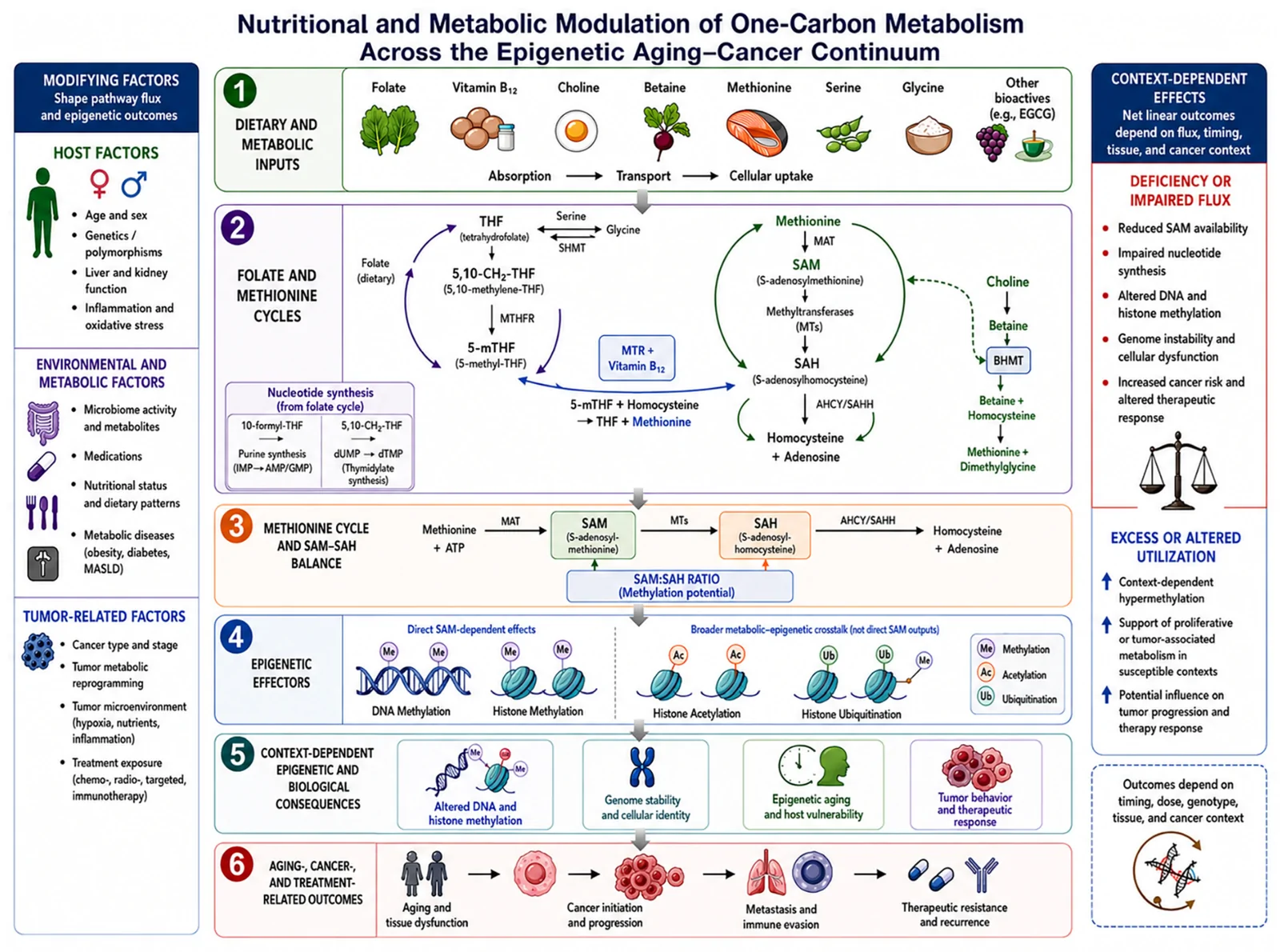
Nutritional and metabolic modulation of one-carbon metabolism across the epigenetic aging–cancer continuum. Dietary folate, vitamin B_12_, choline, betaine, methionine, serine, and glycine contribute to folate and methionine cycles that support nucleotide synthesis and methyl-donor metabolism, whereas selected bioactive compounds may modify metabolic or epigenetic signaling. Folate-derived one-carbon units and betaine support remethylation of homocysteine to methionine, which generates SAM, the principal methyl donor for DNA and histone methylation. Conversion of SAM to SAH influences methylation potential through the SAM:SAH balance. Biological effects vary with nutrient status, metabolic flux, tissue and tumor context, disease stage, and treatment exposure. Solid arrows indicate principal metabolic or biological relationships; dashed arrows indicate indirect or context-dependent relationships. Abbreviations: Ac, acetylation; AHCY/SAHH, S-adenosylhomocysteine hydrolase; AMP, adenosine monophosphate; ATP, adenosine triphosphate; BHMT, betaine–homocysteine S-methyltransferase; dTMP, deoxythymidine monophosphate; dUMP, deoxyuridine monophosphate; EGCG, epigallocatechin gallate; GMP, guanosine monophosphate; IMP, inosine monophosphate; MASLD, metabolic dysfunction-associated steatotic liver disease; Me, methylation; MTHFR, methylenetetrahydrofolate reductase; MTR, methionine synthase; MTs, methyltransferases; 5,10-CH_2_-THF, 5,10-methylenetetrahydrofolate; 5-mTHF, 5-methyltetrahydrofolate; SAH, S-adenosylhomocysteine; SAM, S-adenosylmethionine; SHMT, serine hydroxymethyltransferase; THF, tetrahydrofolate; Ub, ubiquitination.

One-carbon metabolism integrates the folate and methionine cycles with amino-acid metabolism, nucleotide synthesis, redox regulation, and methylation reactions. Folate, vitamins B6 and B12, choline, betaine, methionine, serine, and glycine contribute directly or indirectly to one-carbon transfer and SAM production. The resulting SAM:SAH ratio is influenced not only by dietary intake but also by homocysteine clearance, cellular proliferation, renal and hepatic function, inflammation, and tissue-specific metabolic demand [133]. Figure 1 summarizes how these nutritional and metabolic inputs converge on one-carbon flux, SAM–SAH balance, epigenetic regulation, and context-dependent biological outcomes.

Disruption of one-carbon metabolism can compromise genome stability through several mechanisms. Folate or vitamin B12 deficiency may impair nucleotide synthesis, promote uracil misincorporation into DNA, disturb homocysteine metabolism, and alter methylation potential [134]. These effects may contribute to DNA damage and aberrant DNA or histone methylation.

However, the relationship is not uniformly protective. Proliferating malignant cells also depend on one-carbon metabolism for nucleotide production, redox homeostasis, and methylation reactions. Increasing methyl-donor or one-carbon nutrient availability may therefore have different consequences before malignant transformation, during early tumor development, and after cancer has become established [135]. The biological effect depends on whether the intervention corrects a deficiency, modifies physiological nutrient availability, or produces supraphysiological exposure.

Aging further modifies one-carbon metabolism through changes in dietary intake, gastrointestinal absorption, renal and hepatic function, medication use, and enzyme activity [136]. These variables may contribute to interindividual differences in methylation profiles and blood-derived epigenetic-aging measures. Associations among folate-related metabolites, homocysteine, dietary patterns, and epigenetic clocks have been reported, but they remain heterogeneous and are affected by blood-cell composition, smoking, adiposity, kidney function, supplement use, socioeconomic factors, and underlying disease [25,137].

Methylation of repetitive elements such as LINE-1 and Alu sequences is often used as an approximate measure of global genomic methylation. Although one-carbon nutrient status may influence these markers, findings differ across tissues, populations, analytical platforms, and nutritional contexts [38,138]. Current evidence does not establish that methyl-donor supplementation reliably prevents age-associated hypomethylation, slows EAA, or reduces cancer risk.

A distinction must therefore be maintained between correcting a documented deficiency and administering supplements above physiological requirements. Restoring inadequate nutrient status may normalize metabolic function, whereas high-dose supplementation may have different or potentially adverse consequences [139]. In people with premalignant lesions or established tumors, interventions affecting one-carbon metabolism should be evaluated according to dose, timing, genotype, nutritional status, tumor type, disease stage, and treatment exposure.

### 4.3. Diet- and Microbiome-Derived Epigenetic Modifiers

Diet-derived bioactive compounds and microbiome-generated metabolites can influence chromatin-regulating enzymes, inflammatory and metabolic signaling, redox homeostasis, and ncRNA expression [140,141]. However, most mechanistic evidence derives from cell and animal models, often using exposures that exceed those achieved through customary dietary intake. These findings demonstrate the metabolic responsiveness of the epigenome but do not establish dietary compounds as clinically effective epigenetic therapies. Table 2 summarizes representative modifiers, mechanisms, evidence levels, and translational limitations.

Plant-derived compounds, including sulforaphane, EGCG, curcumin, and resveratrol, have been linked experimentally to HDAC or DNMT modulation, NRF2- and AMPK-associated signaling, mitochondrial function, inflammatory pathways, and ncRNA regulation [145,146,147,148,149,150,151,152,153,154,155,156,157]. Their pleiotropic actions, limited or variable bioavailability, rapid metabolism, and tissue-specific exposure make it difficult to attribute in vivo effects to a single epigenetic mechanism.

The intestinal microbiome adds another metabolic interface by converting dietary substrates into bioactive metabolites. Fermentation of non-digestible carbohydrates generates short-chain fatty acids, including acetate, propionate, and butyrate, which can influence epithelial metabolism, immune regulation, and chromatin signaling [162]. Butyrate illustrates the reciprocal relationship between metabolism and epigenetic regulation: normal colonocytes efficiently oxidize butyrate, whereas the greater reliance of some colorectal cancer cells on glycolysis can promote intracellular butyrate accumulation and stronger HDAC-inhibitory activity [158]. Thus, tumor metabolic state can determine the epigenetic effect of a microbiome-derived metabolite.

Microbial metabolite production nevertheless varies with microbiome composition, dietary substrate availability, intestinal transit, medications, age, host genetics, geography, and disease state [163]. Similar dietary exposures may therefore produce different metabolite concentrations and epigenetic responses among individuals. Current evidence does not establish that any specific dietary bioactive compound or microbiome configuration reliably slows epigenetic aging or prevents cancer.

### 4.4. Implications for Circulating Biomarkers and Therapeutic Response

Nutritional and metabolic status can influence both the biology captured by circulating biomarkers and the composition of the circulating analyte pool. Obesity, insulin resistance, inflammation, sarcopenia, micronutrient deficiencies, organ dysfunction, and related host factors can modify blood-cell methylation, cfDNA release, EV production, and c-ncRNA profiles [164]. Blood-cell epigenetic-aging measures are additionally influenced by immune-cell composition, smoking, adiposity, and metabolic health [165], whereas c-ncRNAs may originate from malignant and multiple non-malignant tissues [166]. Nutritional and metabolic state can therefore act simultaneously as a biological modifier and a source of biomarker variation, supporting collection of relevant anthropometric, metabolic, medication, and comorbidity data [167].

Metabolic state may also modify therapeutic response. One-carbon metabolism supports nucleotide synthesis, redox homeostasis, and methylation reactions, while acetyl-CoA, NAD^+^, α-ketoglutarate, and related metabolites can influence chromatin states associated with stress adaptation and drug tolerance [168]. Microbiome-derived metabolites may further modify immune signaling and treatment response [169]. These relationships are reciprocal: metabolism can shape epigenetic plasticity during therapy, whereas epigenetic reprogramming can alter metabolic dependencies that support treatment adaptation.

Diet-derived compounds and concentrated supplements may additionally interact with anticancer therapies through drug-metabolizing enzymes, transporters, redox pathways, platelet function, and hepatic metabolism [170,171]. They should therefore not be assumed to be harmless or therapeutically beneficial during treatment, particularly at exposures substantially exceeding customary dietary intake.

Serial circulating-biomarker measurements may help determine whether nutritional or metabolic interventions produce biological effects, but biomarker change should not be equated with patient benefit unless linked prospectively to treatment response, toxicity, recurrence, survival, or quality of life [172]. Interpretation also requires consideration of dietary measurement error, tissue- and cell-composition effects, and reverse causality because cancer itself can alter appetite, metabolism, body composition, and circulating profiles [173,174,175]. Nutrition and microbiome-derived metabolites should therefore be regarded as context-dependent modifiers rather than established determinants of epigenetic aging or therapeutic response. Their reciprocal interaction with epigenetic regulation provides a mechanistic bridge to the therapeutic plasticity and resistance mechanisms discussed in Section 5.

## 5. Epigenetic Plasticity and Therapeutic Response Across the Aging–Cancer Continuum

Therapeutic resistance remains a major obstacle to durable cancer control. Stable genetic alterations can confer resistance by modifying drug targets, activating compensatory pathways, or disrupting DNA-damage responses, but treatment failure may also arise through reversible, non-genetic adaptation [176]. Under therapeutic pressure, a subset of tumor cells may enter a slow-cycling or transiently quiescent drug-tolerant persister (DTP) state characterized by chromatin remodeling, transcriptional plasticity, metabolic adaptation, and enhanced stress tolerance [177]. These cells can survive initial treatment without carrying pre-existing resistance-conferring mutations and may subsequently contribute to tumor regrowth [178].

The biological context in which therapeutic adaptation occurs is also shaped by the host. Age-associated inflammation, senescence, immune dysfunction, and metabolic alterations may influence treatment tolerance, tissue recovery, and the selective environment experienced by residual tumor cells [164,165]. These host factors should not be interpreted as direct causes of resistance, but they can modify therapeutic pressure and complicate interpretation of circulating changes during treatment.

**Figure 2:**
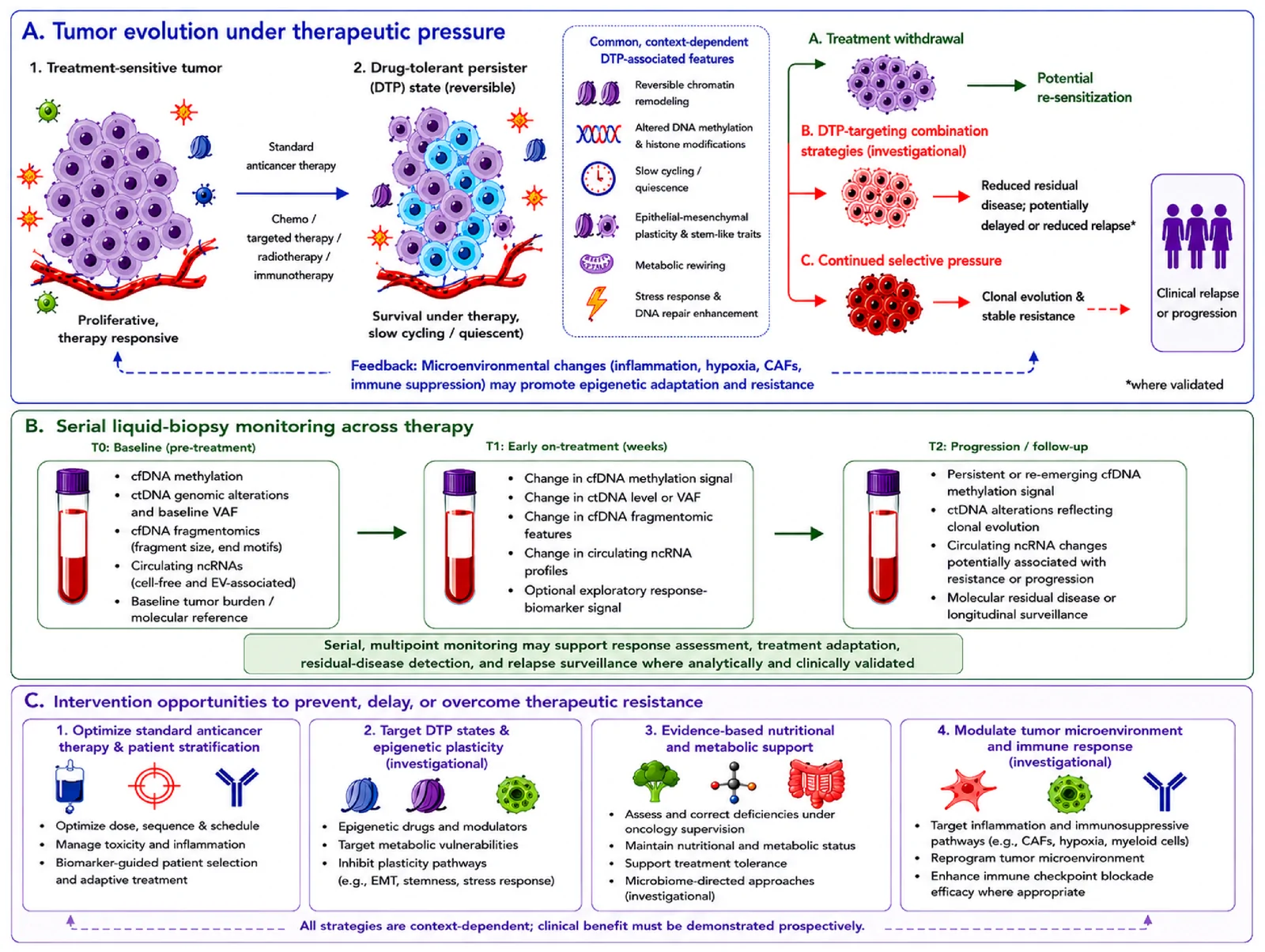
Epigenetic plasticity, circulating biomarker dynamics, and potential intervention opportunities during cancer therapy. Anticancer treatment may eliminate sensitive cells while selecting for or inducing DTP states characterized by reversible chromatin remodeling, phenotypic plasticity, metabolic rewiring, and enhanced stress tolerance. Some DTP cells may regain sensitivity after treatment withdrawal, whereas continued therapeutic pressure can promote stable resistance, clonal evolution, and relapse. Serial liquid-biopsy monitoring may capture changes in cfDNA methylation and fragmentomics, ctDNA genomic alterations, and c-ncRNA profiles and, where clinically validated, support assessment of molecular response, residual disease, and emerging resistance. Potential interventions include optimization of established therapy and investigational targeting of epigenetic, metabolic, or microenvironmental dependencies. Solid arrows indicate principal biological or clinical relationships; dashed arrows indicate indirect, proposed, or context-dependent relationships and feedback loops. Abbreviations: CAFs, cancer-associated fibroblasts; cfDNA, cell-free DNA; ncRNAs, non-coding RNAs; ctDNA, circulating tumor DNA; DTP, drug-tolerant persister; EVs, extracellular vesicles; VAF, variant allele frequency.

DTP states are heterogeneous and vary according to tumor lineage, treatment class, microenvironmental conditions, and duration of exposure. Common features include altered chromatin accessibility and histone modifications, stress-response activation, epithelial–mesenchymal plasticity, stem-like properties, reduced proliferation, and dependence on alternative metabolic pathways [179]. Epigenetic reprogramming can itself reshape glycolytic, mitochondrial, lipid, redox, and other metabolic programs that support survival under therapeutic stress, while altered metabolite availability can reciprocally stabilize adaptive chromatin states. Some DTP cells may regain treatment sensitivity after therapeutic pressure is withdrawn, whereas prolonged persistence can facilitate stable epigenetic reprogramming, clonal selection, or acquisition of resistance-associated mutations [177]. This dynamic transition between reversible tolerance and stable resistance provides the rationale for combining longitudinal molecular monitoring with strategies targeting chromatin plasticity, metabolic dependencies, immune adaptation, and the tumor microenvironment (Figure 2).

### 5.1. Epigenetic and Metabolic Adaptation in Drug-Tolerant Persister State

The DTP model provides a framework for understanding how tumor cells may survive treatment without relying exclusively on pre-existing resistant clones. In the original description, a reversible drug-tolerant subpopulation displayed markedly reduced sensitivity to anticancer agents and depended on an altered chromatin state involving lysine-specific demethylase 5A (KDM5A) [180]. Persister-like phenotypes have subsequently been described across multiple malignancies and treatment classes, although their molecular basis and clinical importance vary considerably [179].

Therapeutic exposure can induce or select chromatin states that reduce dependence on the pathway initially driving tumor growth. Alterations in DNA methylation, histone modifications, enhancer activity, transcription-factor occupancy, and nucleosome organization may facilitate transitions among proliferative, quiescent, invasive, inflammatory, and stem-like states [181]. Because these changes can occur without permanent alteration of the DNA sequence, they provide a relatively rapid route to phenotypic adaptation under therapeutic pressure.

Epithelial–mesenchymal plasticity represents one route through which treatment tolerance may emerge. Partial or reversible mesenchymal programs can reduce proliferation, alter apoptotic thresholds, increase motility, and modify interactions with stromal and immune cells [182]. Stem-like transcriptional states may similarly support self-renewal and tumor repopulation following treatment [183]. These phenotypes exist along dynamic continua rather than as binary states, with their properties shaped by lineage, microenvironment, and therapeutic pressure [184].

Metabolic adaptation is closely integrated with these epigenetic transitions. Depending on tumor type and treatment, DTP cells may reconfigure glycolysis, oxidative phosphorylation, fatty-acid oxidation, amino-acid utilization, autophagy, and redox homeostasis to sustain survival under therapeutic stress [185]. Epigenetic programs can reinforce these metabolic states by altering transcription of metabolic enzymes and stress-response pathways, whereas changes in SAM, acetyl-CoA, NAD^+^, α-ketoglutarate, and FAD availability can reciprocally influence DNA and histone methylation, histone acetylation, and demethylation [122,185]. Mitochondrial function and redox balance are particularly important because they determine both energy production and the availability of metabolites and cofactors required for chromatin regulation. These dependencies may create treatment opportunities, but they are not necessarily tumor-specific and may also be required by activated immune cells or regenerating normal tissues.

Persistence does not inevitably progress to stable resistance [186]. Some DTP cells may be eliminated by continued therapy or immune surveillance, whereas others may regain sensitivity after treatment withdrawal [187]. Prolonged survival under selective pressure can nevertheless permit stable enhancer reprogramming, clonal selection, or acquisition of resistance-associated mutations [188]. DTP states therefore represent a potential intermediate between treatment sensitivity and acquired resistance rather than a universal evolutionary trajectory [189].

### 5.2. Circulating Biomarkers of Treatment Response and Resistance

Therapeutic adaptation is dynamic and spatially heterogeneous, creating a need for biomarkers that can be measured repeatedly during treatment. Liquid biopsy complements tissue analysis by enabling longitudinal assessment of molecular change at baseline, during therapy, and at progression [190].

Mutation-based ctDNA provides highly specific information when trackable tumor-associated variants are available. Declining ctDNA levels may accompany molecular response, whereas persistent or increasing levels can indicate incomplete tumor clearance, progression, or emerging resistance. In selected patients receiving immune-checkpoint inhibitors, longitudinal ctDNA analysis may also help distinguish pseudoprogression from true progression, although this application remains context-dependent [107,191]. cfDNA methylation and fragmentomic approaches provide complementary information without requiring predefined recurrent mutations and may support longitudinal estimation of tumor burden or treatment response [192,193].

c-ncRNAs may capture more rapid transcriptional, immune, and intercellular-signaling changes associated with therapeutic adaptation [194]. EV-associated and non-vesicular miRNAs, lncRNAs, and circRNAs have been linked experimentally to epithelial–mesenchymal plasticity, DNA-damage responses, immune regulation, and chemotherapy resistance [195,196]. However, treatment-associated changes in circulating RNA abundance may also reflect inflammation, platelet activation, immune-cell turnover, tissue injury, metabolic change, or treatment toxicity rather than tumor-specific adaptation [100].

The greatest translational value of serial liquid biopsy may therefore lie in combining complementary molecular signals with imaging, pathological response, treatment exposure, and relevant host variables to identify emerging resistance early enough to influence management [197]. Multianalyte approaches should nevertheless demonstrate incremental value over simpler assays and established clinical predictors. Until prospective studies show that biomarker-guided treatment modification improves outcomes within a defined clinical setting, these approaches should remain investigational monitoring tools rather than stand-alone determinants of therapy.

### 5.3. Nutritional and Metabolic Modulation as an Investigational Adjunct

The metabolic dependence of epigenetic regulation has stimulated interest in nutritional and metabolic interventions as adjuncts to established cancer therapy [198]. These approaches aim to modify systemic nutrient availability, growth-factor signaling, immune metabolism, or tumor-specific metabolic dependencies. They should not be presented as methods for resetting epigenetic clocks or reversing biological aging, because such outcomes have not been established in patients with cancer [199].

Fasting and fasting-mimicking interventions have been investigated as strategies for transiently reducing glucose, insulin, insulin-like growth factor 1 (IGF-1), and amino-acid signaling during treatment [200]. Preclinical studies suggest that fasting-associated metabolic states may modify stress responses in malignant and non-malignant cells [201,202]. Early clinical studies indicate that cyclic fasting-mimicking interventions can be feasible in carefully selected patients and can produce measurable metabolic and immunological changes [203,204]. These findings establish biological activity and feasibility rather than therapeutic efficacy; evidence for improved progression-free or overall survival remains insufficient and may depend on baseline nutritional status, body composition, tumor type, treatment regimen, and adherence.

Methionine restriction is mechanistically relevant because methionine contributes to protein synthesis, SAM production, nucleotide metabolism, redox homeostasis, and tumor-cell proliferation. Dietary methionine restriction has enhanced the effects of radiotherapy or systemic treatment in experimental models [205]. A phase I pilot study demonstrated the feasibility of a methionine-restricted diet during definitive radiotherapy in a small and clinically heterogeneous cohort [206]. This provides proof of feasibility but not evidence of clinical efficacy or a basis for routine patient selection.

Metabolic modulation may also be achieved pharmacologically by inhibiting one-carbon metabolism, oxidative phosphorylation, fatty-acid oxidation, NAD^+^ metabolism, or redox-buffering pathways. Such approaches may exploit dependencies associated with DTP or resistant states [207]. More specifically, increased fatty-acid oxidation can support DNA-damage repair and redox homeostasis in some tumor contexts, thereby contributing to resistance to genotoxic anticancer therapies [207]. Because these pathways also support activated immune cells and regenerating normal tissues, therapeutic targeting may impair antitumor immunity, treatment tolerance, or tissue recovery [208].

Nutritional interventions require particular caution because many patients are already vulnerable to weight loss, sarcopenia, cachexia, micronutrient deficiency, and gastrointestinal toxicity [209,210]. Restrictive regimens should therefore be evaluated under medical and dietetic supervision. In many settings, correction of documented deficiencies and preservation of lean body mass may be more clinically relevant than unvalidated dietary restriction [211].

Nutritional and metabolic strategies should therefore be regarded as investigational modifiers of tumor and host metabolism rather than substitutes for established anticancer treatment. Their translation requires appropriate patient selection, safe and biologically effective exposure, pharmacodynamic confirmation, and prospective evidence that metabolic or biomarker changes translate into improved clinical outcomes.

### 5.4. Epigenetic Drugs and Mechanism-Based Combination Strategies

Epigenetic drugs target enzymes or regulatory proteins involved in chromatin organization and transcriptional control. Table 3 summarizes the principal biomarker and intervention strategies.

Clinically established epigenetic therapies include DNA methyltransferase inhibitors (DNMTis), such as azacitidine and decitabine, and histone deacetylase inhibitors (HDACis), such as vorinostat, romidepsin, belinostat, and panobinostat. These agents have demonstrated clinical activity in selected hematological malignancies, whereas their efficacy as monotherapies in most solid tumors has been limited by tumor heterogeneity, incomplete target specificity, toxicity, pharmacokinetic constraints, and adaptive chromatin plasticity. Selective targeting of PRC2/EZH2 has provided an additional clinically validated epigenetic strategy in defined molecular contexts.

The deoxyribonucleotide metabolites of azacitidine and decitabine become incorporated into DNA and covalently trap DNMTs, leading to enzyme depletion and dose- and schedule-dependent effects on DNA methylation, differentiation, immune signaling, and cell viability [234]. Decitabine is incorporated primarily into DNA, whereas azacitidine is incorporated predominantly into RNA, with a smaller fraction converted into a deoxyribonucleotide form that enters DNA [235]. At lower, less cytotoxic exposures, these agents may promote hypomethylation, re-expression of selected genes, and altered antigen presentation, whereas higher exposures increasingly produce direct cytotoxic effects [236].

HDACis affect histone and non-histone substrates and should not be viewed simply as agents that globally open chromatin. Their effects extend to transcription, protein stability, DNA repair, cell-cycle regulation, apoptosis, and immune signaling. This pleiotropic activity contributes to both their therapeutic potential and dose-limiting toxicity [237].

Beyond DNMTis and HDACis, PRC2 has emerged as a clinically relevant epigenetic target. EZH2 is the principal catalytic subunit of PRC2 and mediates H3K27 trimethylation, thereby reinforcing transcriptional repression at selected loci [238]. Genetic or functional dependence on EZH2 occurs in defined malignancies, including follicular lymphoma and diffuse large B-cell lymphoma harboring activating EZH2 mutations, as well as SMARCB1-deficient epithelioid sarcoma [239,240]. Pharmacological EZH2 inhibition reduces H3K27me3 and can derepress PRC2-regulated transcriptional programs in susceptible tumors [239]. Tazemetostat, an oral selective EZH2 inhibitor, has shown clinical activity in relapsed or refractory follicular lymphoma and advanced epithelioid sarcoma, providing proof of principle that Polycomb-dependent chromatin states can be therapeutically exploited [240,241]. However, sensitivity to EZH2 inhibition depends strongly on tumor lineage, genetic background, and chromatin context, and resistance or limited activity in some settings supports continued development of biomarker-guided combinations and next-generation PRC2-targeting strategies [238].

Mechanism-based combinations aim to exploit epigenetic reprogramming while overcoming the limited activity of epigenetic-drug monotherapy [35]. Potential partners include chemotherapy, targeted agents, immune-checkpoint inhibitors, DNA-damage-response inhibitors, apoptosis-regulating agents, and metabolic therapies. Depending on the context, the rationale may involve enhanced tumor-antigen expression, disruption of adaptive chromatin states, restoration of apoptotic competence, increased treatment sensitivity, or targeting of dependencies that emerge in DTP cells [242].

Diet-derived bioactive compounds and microbiome-derived metabolites can modulate chromatin and signaling pathways experimentally, but combinations with DNMTis or HDACis remain predominantly preclinical [243]. Because customary dietary exposure may not reproduce concentrations used experimentally and concentrated supplements may alter drug metabolism, transport, toxicity, or treatment efficacy, proposed combinations require direct pharmacokinetic, pharmacodynamic, safety, and efficacy evidence [244,245].

### 5.5. Translational Challenges and Clinical Trial Priorities

Clinical translation requires careful attention to intervention timing, patient selection, biomarker context, and clinically meaningful endpoints. Because therapeutic adaptation evolves during treatment, baseline measurements alone may not capture the DTP or resistant states that subsequently emerge. Trials should incorporate longitudinal sampling at predefined time points linked to treatment exposure, imaging, pathological response, toxicity, and clinical outcomes [246].

Studies combining epigenetic drugs with targeted, metabolic, immune, or nutritional interventions should be based on a defined mechanistic hypothesis and should specify the expected pharmacodynamic effect. Treatment sequence may be important because chromatin priming, metabolic inhibition, immune activation, and cytotoxicity may have different optimal schedules. Biomarker-defined enrichment may also be necessary because only a subset of tumors is likely to depend on a particular chromatin regulator or metabolic vulnerability [247].

Circulating biomarkers should be interpreted alongside clinical and physiological data because treatment-associated changes in cfDNA or c-ncRNAs may reflect tumor response, host injury, hematopoietic recovery, or several processes simultaneously [248,249]. This distinction matters when biomarkers identify adaptive or resistant states rather than simply estimating tumor burden.

Prospective trials should determine whether biomarker-guided treatment decisions improve outcomes beyond standard management; Section 7.2 discusses the broader requirements for clinical validation and utility. Taken together, epigenetic and metabolic plasticity can support reversible drug tolerance and the evolution of stable therapeutic resistance. Translation therefore requires longitudinal, mechanism-informed studies that identify adaptive states during therapy and test whether biomarker-guided epigenetic or metabolic interventions improve clinically meaningful outcomes in appropriately selected patients.

## 6. Technological Frontiers for Resolving the Epigenetic Aging–Cancer Continuum

Understanding the epigenetic aging–cancer continuum requires technologies that can resolve cellular heterogeneity, distinguish tumor-derived from host-derived molecular signals, and integrate epigenetic information with nutritional, metabolic, microbial, and clinical variables. Conventional bulk profiling remains valuable for identifying population-level patterns, but it averages signals across heterogeneous cell populations and may obscure rare or transitional states, including DTP cells, cancer stem-like populations, and distinct immune or stromal compartments [3,250].

**Figure 3:**
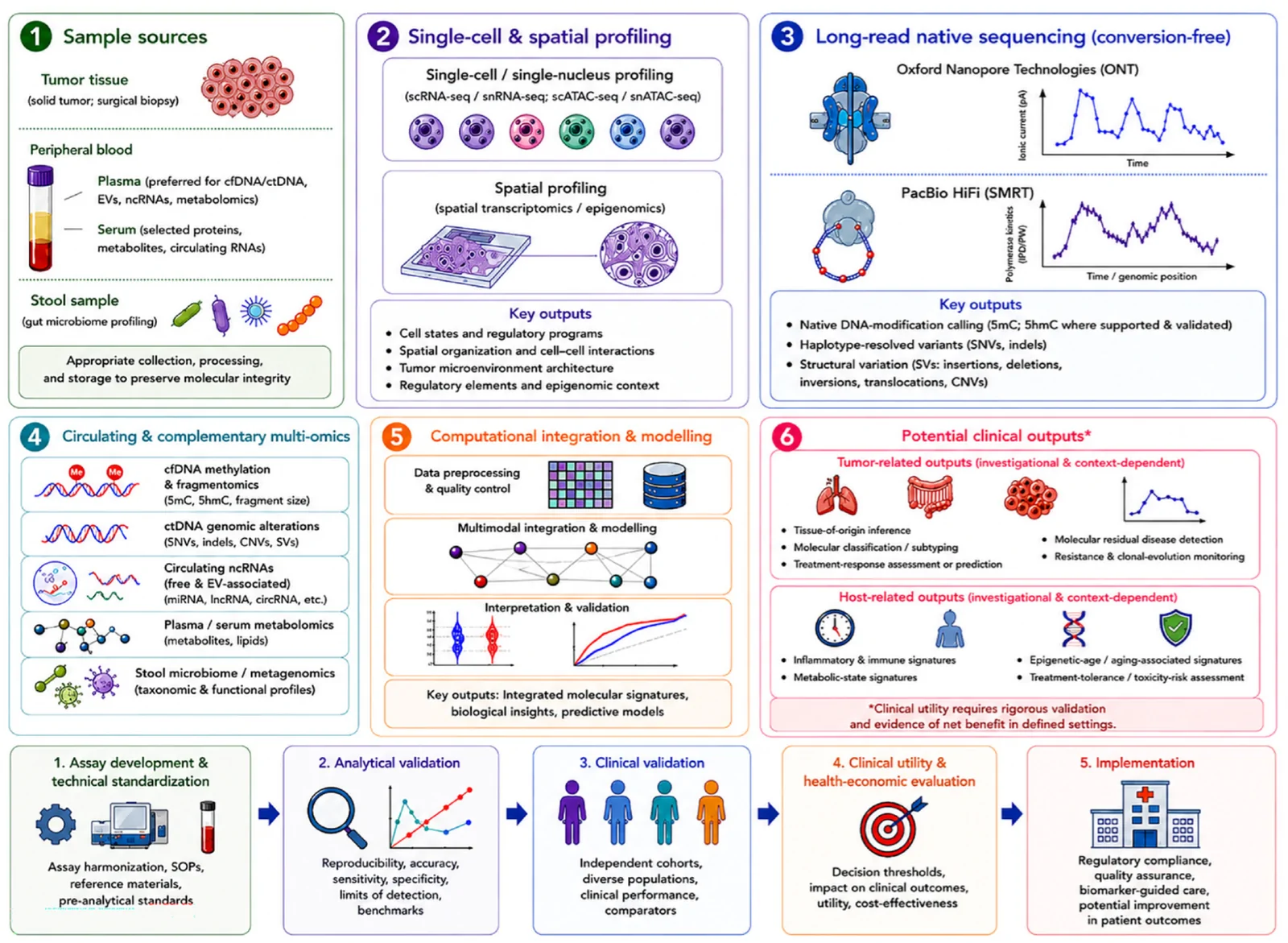
Multi-scale technological integration to resolve the epigenetic aging–cancer continuum and support clinical translation. Complementary single-cell, single-nucleus, spatial, native long-read, circulating, and microbiome-based multi-omic approaches can interrogate tumor tissue, blood, and stool. Single-cell and single-nucleus profiling resolves cellular states and regulatory programs, whereas spatial profiling preserves tissue architecture and microenvironmental relationships. Native long-read sequencing enables DNA-modification analysis together with haplotype and structural-variant information. Circulating multi-omic profiling can integrate cfDNA methylation and fragmentomics, ctDNA genomic alterations, c-ncRNAs, metabolomics, and microbiome features. Computational integration may identify signatures relevant to tumor classification, tissue of origin, treatment response, MRD, resistance, and host biological state. Clinical translation requires standardized assays, validation, and demonstration of utility within a defined context of use. Solid arrows in the translational pathway indicate progression from assay development through validation to clinical implementation. Abbreviations: cfDNA, cell-free DNA; circRNA, circular RNA; ctDNA, circulating tumor DNA; EVs, extracellular vesicles; HiFi, high-fidelity sequencing; lncRNA, long non-coding RNA; miRNA, microRNA; ncRNAs, non-coding RNAs; ONT, Oxford Nanopore Technologies; PacBio, Pacific Biosciences; scATAC-seq, single-cell assay for transposase-accessible chromatin sequencing; scRNA-seq, single-cell RNA sequencing; SMRT, single-molecule real-time sequencing; snATAC-seq, single-nucleus assay for transposase-accessible chromatin sequencing; snRNA-seq, single-nucleus RNA sequencing; SNV, single-nucleotide variant; SV, structural variant; 5hmC, 5-hydroxymethylcytosine; 5mC, 5-methylcytosine.

Single-cell and single-nucleus approaches can resolve cell-type-specific transcriptional and epigenetic states, whereas spatial profiling preserves tissue architecture and enables examination of these states within their anatomical and tumor-microenvironment context. Native long-read sequencing can extend this resolution by interrogating DNA modifications, haplotypes, repetitive elements, and structurally complex genomic regions. Multimodal computational integration can then connect these tissue-level observations with circulating biomarkers, metabolic profiles, microbiome features, and host-related variables [251,252,253]. The principal value of these approaches lies in revealing biologically informative cell states, lineage-specific alterations, spatial niches, and adaptive populations that bulk measurements may obscure, and in translating these discoveries into simpler, scalable, clinically interpretable assays. Figure 3 summarizes this multi-scale framework.

### 6.1. Single-Cell, Single-Nucleus, and Spatial Profiling

Single-cell and single-nucleus approaches resolve cell-type-specific transcriptional, chromatin-accessibility, and DNA-methylation states that are obscured by bulk profiling. scATAC-seq and scRNA-seq provide complementary information on regulatory-element activity and its transcriptional consequences, whereas integrated and methylation-based approaches can further define lineage and cell-state heterogeneity [254,255].

Importantly, recent studies show that this increased resolution yields biologically informative observations relevant to aging, not just greater technical granularity. Single-cell transcriptional and chromatin-accessibility profiling of human peripheral blood immune cells across the lifespan revealed age-associated reprogramming across both lymphoid and myeloid lineages, with particularly marked transcriptional remodeling of T cells and an increase in *IL1B*^hi^ monocytes with aging, linking cell-specific molecular changes to inflammaging [255]. These findings illustrate that age-associated epigenetic remodeling is not uniform across cell populations and are consistent with broader evidence that aging progressively alters cellular identity and regulatory state [256].

Single-cell profiling has likewise provided direct evidence of treatment-associated lineage plasticity in cancer. In transformed small-cell lung cancer, single-cell analysis revealed increased intratumoral heterogeneity and identified a recurrent stem-like malignant population associated with lineage transformation; interferon-stimulated gene-positive T and B cells were also enriched in transformed tumors, with experimental evidence linking type I interferon signaling to neuroendocrine differentiation [257]. Together with broader evidence connecting epithelial–mesenchymal plasticity and tumor-intrinsic transcriptional programs with epigenetic heterogeneity and microenvironmental remodeling [258,259], these observations show how single-cell approaches can identify adaptive cell states and regulatory programs that may be diluted in bulk tissue. High-coverage single-cell DNA methylation profiling can further resolve lineage- and allele-specific methylation states, providing a route to refine candidate epigenetic biomarkers identified by bulk assays [260].

Spatial transcriptomic and epigenomic approaches complement these measurements by retaining anatomical context. Unlike dissociation-based single-cell workflows, many spatial approaches analyze intact tissue sections and therefore preserve tissue architecture and local cell–cell relationships. They can map malignant, immune, stromal, senescent, and vascular programs to hypoxic, invasive, immune-excluded, or treatment-remodeled niches, allowing molecular cell states to be interpreted within the microenvironment in which they arise [261]. This is particularly relevant to the aging–cancer continuum, in which senescence, inflammation, immune dysfunction, and therapeutic adaptation are strongly shaped by local cellular interactions.

Platform-specific limitations remain: dissociation-based workflows can induce stress responses or underrepresent fragile populations, whereas single-nucleus approaches reduce some of these effects but provide less information on cytoplasmic processes [262,263]. Nevertheless, the main value of high-resolution profiling in this context is biological discovery—identifying cell states, regulatory programs, spatial niches, and candidate biomarkers that can subsequently be validated using simpler, clinically scalable assays.

### 6.2. Native Long-Read Epigenetic Analysis

Conventional bisulfite and short-read sequencing remain widely used for DNA-methylation profiling but have limitations in repetitive and structurally complex genomic regions and can introduce DNA loss, reduced sequence complexity, and mapping bias during chemical conversion [264]. These constraints are relevant to the aging–cancer continuum because repetitive elements undergo substantial epigenetic remodeling. For example, genome-wide analysis of blood cfDNA identified age-associated hypomethylation of LINE-1 and Alu retroelements, supporting repetitive-element methylation as a measurable component of human epigenetic aging [265]. Conventional bisulfite sequencing also does not distinguish 5-methylcytosine (5mC) from 5-hydroxymethylcytosine (5hmC) without additional chemical or enzymatic procedures.

Native long-read sequencing provides a complementary approach by combining sequence and DNA-modification information without bisulfite conversion [266]. Oxford Nanopore Technologies (ONT) infers sequence and selected base modifications from nanopore ionic-current signals [267], whereas PacBio HiFi combines highly accurate long reads with modification inference from polymerase-associated kinetic information [268]. Both approaches can support haplotype phasing, structural-variant analysis, and modification profiling, although their analytical characteristics differ.

The biological advantage is the ability to connect genetic and epigenetic information on individual molecules. Long-read analysis can resolve allele-specific methylation, imprinting, repetitive elements, and structurally complex loci that are difficult to interrogate with short reads [269,270]. As a proof of principle, long-read sequencing has resolved imprinted methylation together with the corresponding allelic context, demonstrating how methylation states can be assigned to specific haplotypes rather than averaged across genomic copies [270]. Such molecule-resolved information could help determine whether aging- or cancer-associated epigenetic alterations arise on particular alleles, repetitive elements, or structurally altered genomic backgrounds.

For liquid biopsy, native sequencing avoids conversion-associated DNA loss but does not overcome the biological fragmentation of plasma cfDNA or the low abundance of tumor-derived molecules [269,271]. DNA-input requirements, sequencing depth, modification-calling accuracy, cost, and interlaboratory reproducibility therefore remain important considerations [272]. Native long-read approaches are therefore most valuable for mechanistic discovery and resolving complex epigenetic states, whereas targeted short-read or enzymatic assays may remain preferable for scalable clinical testing.

### 6.3. Multimodal Integration and Interpretable Machine Learning

Circulating epigenetic studies increasingly combine cfDNA methylation, ctDNA mutations, fragmentomic features, c-ncRNAs, metabolites, microbiome profiles, nutritional exposures, imaging, and clinical variables. The value of multimodal integration lies not in increasing molecular complexity per se, but in determining whether complementary signals improve biological interpretation or clinical performance beyond individual analytes [192,273].

DNA methylation is particularly informative for tissue-of-origin and cell-type deconvolution because methylation patterns are closely linked to cellular identity. Reference methylome atlases can estimate the tissues and cell populations contributing to the cfDNA pool and help infer the origin of cancer-associated signals [74,274]. Accuracy nevertheless depends on tumor fraction, reference-atlas completeness, and retention of lineage-associated methylation programs. Aging, inflammation, dedifferentiation, metastasis, and treatment-associated remodeling can alter these programs, supporting the inclusion of biologically diverse reference states where feasible [275,276].

A central objective of multimodal modeling is to distinguish tumor-derived biology from host-related variation. Chronological age, inflammation, blood-cell composition, metabolic disease, nutritional status, treatment exposure, and technical batch effects can contribute substantially to predictive signatures and may themselves carry biologically or clinically relevant information [11,277]. Their contributions should therefore be resolved explicitly rather than interpreted as tumor specificity.

Model complexity should be matched to cohort size, missingness, interpretability, and the intended clinical question [278]. In high-dimensional datasets with limited patient numbers, regularized or ensemble approaches may perform comparably to more complex architectures while remaining easier to interpret and validate [279,280]. Additional molecular, nutritional, or metabolic variables should therefore be incorporated only when they provide reproducible incremental information beyond established predictors. Feature-attribution methods may identify influential variables but do not establish causality. Section 7 addresses detailed requirements for model validation, calibration, reproducibility, and clinical utility.

Table 4 summarizes the principal outputs, biological contributions, limitations, and translational maturity of the technologies discussed in this section.

Taken together, single-cell and spatial profiling, native long-read sequencing, and interpretable multimodal integration can resolve cell states, tissue context, tumor–host contributions, and adaptive trajectories that are obscured by individual assays. Their principal translational value may lie in identifying biologically informative signals that can subsequently be reduced to focused, scalable, and clinically interpretable biomarker strategies. Section 7 addresses broader requirements for analytical validity, causal inference, reproducibility, and clinical utility.

## 7. Translational Bottlenecks, Methodological Challenges, and Causal Inference

Despite strong biological links among nutritional and metabolic exposures, epigenetic aging, circulating biomarkers, and cancer, translation into clinically useful tools remains challenging. Performance often declines outside discovery cohorts because of pre-analytical variation, exposure misclassification, biological heterogeneity, confounding, reverse causality, overfitting, and differences between development cohorts and intended-use populations. Translation therefore requires progression from analytical validity and biological plausibility to independent clinical validation and, ultimately, evidence that biomarker-guided management improves a defined clinical decision and patient-relevant outcomes. Table 5 summarizes the principal sources of bias and corresponding mitigation strategies.

### 7.1. Measurement Validity, Biological Heterogeneity, and Causal Inference

Circulating-biomarker measurements are sensitive to biospecimen handling. Delayed plasma separation, leukocyte lysis, hemolysis, platelet contamination, repeated freeze–thaw exposure, and assay-specific effects can alter cfDNA, EV, or c-ncRNA measurements and should therefore be controlled through standardized procedures and predefined QC criteria [20,66,311,312]. Conversion-based methylation assays may introduce DNA loss and mapping bias, mutation-based ctDNA assays must account for sequencing artifacts and clonal hematopoiesis, and c-ncRNA studies require validated extraction and normalization procedures [271,299]. Table 5 summarizes these analytical considerations, which should be distinguished from genuine biological variation.

Nutritional exposure presents an additional measurement challenge because reported intake may not reflect the concentration of bioactive metabolites reaching a particular tissue. Dietary assessment is affected by recall and portion-estimation error, whereas individual metabolite measurements can vary with recent intake, organ function, medications, microbiome composition, and disease [313]. Repeated dietary assessment combined with objective biomarkers, metabolomics, supplement records, and body-composition measures can reduce misclassification, while sampling should reflect the biological timescale of the process under investigation.

Biological heterogeneity further complicates interpretation. Age, genotype, adiposity, physical activity, organ function, medications, microbiome composition, cancer type, treatment exposure, inflammation, and leukocyte composition can influence metabolic and epigenetic measurements independently of the biological process of interest. Consequently, observed differences in bulk methylation or RNA profiles may reflect altered cellular composition rather than molecular reprogramming within individual cells.

Associations among nutrition, epigenetic aging, circulating biomarkers, and cancer therefore do not establish causation. Confounding and reverse causality are particularly important because preclinical or established cancer can alter appetite, body composition, metabolism, inflammation, microbiome composition, and circulating molecular profiles before diagnosis. An association between EAA and subsequent cancer detection, for example, may reflect occult disease or shared underlying biology rather than a causal effect of accelerated epigenetic aging on tumor initiation. Prospective sampling, repeated measurements, lagged analyses, negative controls, and triangulation across complementary study designs can strengthen causal interpretation.

MR may provide additional evidence using inherited genetic variants, including mQTLs, as instruments [314]. However, valid inference requires appropriate instrument strength and assumptions concerning confounding and horizontal pleiotropy. Linkage disequilibrium, weak instruments, population stratification, tissue mismatch, and lack of colocalization can bias estimates [314]. Sensitivity analyses, bidirectional MR, multiple independent instruments, and formal colocalization should therefore be used where appropriate [305]. Tissue and temporal specificity are particularly important because an mQTL identified in blood may not reproduce the same methylation state in the tissue from which a tumor develops [315]. Robust causal conclusions will therefore require convergence across prospective cohorts, genetic analyses, longitudinal molecular profiling, experimental models, and intervention studies.

### 7.2. Clinical Validation, Reproducibility, and Utility

Clinical validation must occur within a predefined context of use, such as risk assessment, early detection, prognosis, treatment monitoring, MRD surveillance, or resistance detection; evidence supporting one application should not automatically be extrapolated to another [316]. Development and validation populations should reflect the disease prevalence, stage distribution, comorbidity burden, demographic characteristics, and diagnostic pathway of the intended clinical setting [317].

Epigenetic-aging measures illustrate the distinction between biological association and clinical utility. Changes in an epigenetic clock may provide exploratory or mechanistic information but should not be interpreted as reduced cancer risk, improved treatment response, biological rejuvenation, or clinical benefit [318]. Qualification as a surrogate endpoint would require evidence that intervention-induced biomarker changes reliably predict changes in relevant clinical outcomes. At present, epigenetic clocks are not validated surrogate endpoints for cancer incidence, recurrence, progression-free survival, or overall survival [319]. The same principle applies to nutritional and metabolic interventions: changes in SAM:SAH balance, inflammatory markers, c-ncRNA profiles, EAA, or locus-specific methylation may demonstrate biological activity without demonstrating patient benefit [140].

Computational biomarker models likewise require rigorous separation of development and validation. Preprocessing, imputation, feature selection, and model tuning should be confined to training data, and patient- or batch-level information leakage must be prevented [320]. Final models should be locked before independent evaluation, with reporting of discrimination, calibration, decision thresholds, missing-data handling, subgroup performance, and comparison with established clinical predictors [321]. Additional molecular complexity is justified only when it provides reproducible incremental performance.

Representative recruitment is equally important because epigenetic profiles, metabolic health, dietary exposures, microbiome composition, and cancer outcomes vary across demographic and clinical populations. Validation should therefore include clinically representative cohorts and appropriate subgroup analyses. Metadata collection should prioritize variables that materially influence the biomarker or outcome, including smoking, body composition, medications, organ function, inflammatory and metabolic status, treatment exposure, biospecimen handling, and relevant dietary or microbiome variables.

Ultimately, the translational challenge is to identify signals that are analytically reliable, biologically interpretable, clinically informative, and actionable. Clinical utility requires prospective evidence that a biomarker improves a defined decision beyond existing practice, with predefined thresholds and comparison against standard management. Prognostic association alone is insufficient unless biomarker-guided care provides incremental value and improves clinically meaningful outcomes.

## 8. Future Directions: From Mechanistic Integration to Clinical Utility

The convergence of biogerontology, nutritional biochemistry, cancer epigenetics, and precision oncology creates opportunities to develop biomarkers that capture both tumor evolution and host biological state. Future research should, however, be organized around clearly defined clinical questions rather than increasing molecular complexity without a specified context of use. Priority should be given to prospective, longitudinal, mechanism-informed studies that distinguish tumor-derived from host-derived signals and determine whether integrating epigenetic, metabolic, nutritional, and circulating information provides incremental value beyond established clinical predictors.

Prospective intervention studies are particularly needed because much of the evidence linking nutrition, metabolism, epigenetic aging, and cancer remains observational or preclinical. Candidate approaches may include correcting documented nutrient deficiencies, modifying dietary patterns, supervised fasting-mimicking interventions, methionine restriction, fermentable-fiber strategies, or microbiome-directed interventions. Their evaluation should be guided by biological rationale, cancer and treatment context, baseline nutritional and metabolic status, safety, and clinically meaningful endpoints. Epigenetic-aging measures such as PhenoAge [9] and GrimAge [10] may be incorporated as exploratory or mechanistic outcomes, but, as discussed in Section 7.2, changes in these measures should not be interpreted as therapeutic benefit unless prospectively linked to patient-relevant outcomes. Biomarkers proposed for nutritional-response assessment, treatment selection, MRD surveillance, or resistance monitoring likewise require qualification within their intended context of use.

Longitudinal sampling will be essential for separating tumor evolution from host physiological responses. Biospecimens collected before treatment, during therapy, at response assessment, and during follow-up or progression can help distinguish molecular response, reversible adaptation, emerging resistance, treatment toxicity, and host recovery. Collection time, fasting status, recent physical activity, sleep, medication use, and treatment timing can also influence metabolic, immune, and circulating molecular measurements and should therefore be standardized or recorded where biologically relevant [322,323,324]. A central objective should be to determine when host-derived signals represent confounding and when they provide complementary information on treatment tolerance, toxicity, recovery, or systemic vulnerability.

Multimodal precision-oncology strategies should similarly be developed to improve specific clinical decisions rather than maximize the number of measured variables. Integration of circulating epigenetic signals with tumor genomics, imaging, pathology, metabolic and nutritional information, treatment exposure, and conventional clinical predictors may improve biological interpretation and patient stratification [325]. Emerging single-cell, spatial, and long-read technologies will be particularly valuable for identifying the cellular, spatial, and molecular origins of candidate biomarkers, but their principal role may remain discovery and biomarker refinement; scalable implementation will often require simpler and lower-cost assays. Additional modalities should therefore be incorporated only when they provide reproducible incremental information beyond established predictors.

The translational pathway should progress from mechanistic support and analytical validity to independent clinical validation, prospective biomarker-guided evaluation, and demonstration of patient benefit, with the methodological requirements detailed in Section 7 [306,307,326]. International harmonization of biospecimen handling, assay reporting, metadata collection, and computational benchmarking can facilitate this progression but cannot substitute for evidence of clinical utility. Future progress will ultimately depend on studies that jointly resolve tumor and host biology and establish whether this integrated information improves clinically meaningful decisions and outcomes.

## 9. Conclusions

Aging and cancer intersect through progressive changes in genome regulation, cellular identity, metabolism, inflammation, senescence, and tissue homeostasis. Age-associated epigenetic remodeling is not equivalent to malignant transformation, but it can create regulatory vulnerabilities that cancer subsequently exploits to support plasticity, clonal evolution, immune evasion, metastasis, and therapeutic resistance.

Nutrition and metabolism modify this continuum through one-carbon metabolism, acetyl-CoA and NAD^+^ availability, redox regulation, chromatin-modifying enzymes, inflammatory signaling, and microbiome-derived metabolites. Epigenetic reprogramming can reciprocally reshape metabolic pathways that support tumor adaptation and drug tolerance. These mechanisms provide a biological basis for nutrient-sensitive modulation of the aging–cancer continuum but do not establish specific foods, supplements, or restrictive dietary interventions as strategies to reverse epigenetic aging, prevent cancer, or overcome therapeutic resistance.

Circulating biomarkers offer a complementary way to interrogate tumor and host biology. Mutation-based ctDNA, cfDNA methylation, fragmentomics, and c-ncRNAs can capture distinct aspects of tumor burden, origin, evolution, and treatment response, whereas blood-cell DNA methylation clocks primarily reflect host biological state. Their interpretation therefore requires separation of tumor-derived signals from variation associated with aging, inflammation, metabolism, nutrition, and treatment exposure.

Emerging single-cell, spatial, long-read, and multimodal approaches can improve mechanistic resolution and identify candidate biomarkers, but clinical translation ultimately depends on analytically robust assays, representative populations, independent validation, and evidence of incremental clinical utility. The central challenge is therefore not to generate additional molecular signals but to determine which are biologically interpretable, clinically actionable, and capable of improving patient management. The highest research priority is therefore to conduct prospective longitudinal studies that distinguish tumor-derived from host-derived epigenetic signals and determine whether biomarker-guided interventions improve clinically meaningful patient outcomes.

## Figures and Tables

**Table 1:** Comparative characteristics and clinical relevance of circulating biomarker classes and blood-cell aging measures across the epigenetic aging–cancer continuum.

Analyte Class [Refs]	Predominant Source and Principal Biological Information	Influence of Aging, Nutrition, Metabolism, and Treatment	Principal Strengths	Main Limitations	Potential Clinical Applications
cfDNA methylation [57,58,59]	Mixed hematopoietic, tissue-derived, and tumor-derived DNA; cancer-associated regulatory alterations, cell identity, and tissue-of-origin information	Background methylation may be influenced by age-related epigenetic drift, immune-cell composition, inflammation, tissue injury, metabolic disease, nutritional status, and treatment exposure.	Numerous informative genomic regions; preservation of lineage-associated methylation patterns; does not require a predefined tumor mutation	Low tumor fraction, background from non-malignant tissues, DNA loss and bias in workflows, sequencing depth, computational complexity, and dependence on reference populations	Early detection, tissue localization, molecular classification, prognosis, treatment monitoring, and MRD assessment
Mutation-based ctDNA [60,61,62]	Predominantly tumor-derived DNA carrying somatic variants and reflecting clonal evolution	Age primarily affects interpretation through clonal hematopoiesis and altered background cell turnover; nutritional and metabolic effects are generally indirect rather than analyte-specific	High molecular specificity; identification of actionable and resistance-associated variants; compatibility with tumor-informed monitoring	Limited abundance in early-stage or low-shedding disease; tumor heterogeneity; limited number of trackable variants; clonal hematopoiesis; sampling and sequencing-depth requirements	Molecular genotyping, targeted-therapy selection, treatment-response assessment, resistance monitoring, and MRD surveillance
cfDNA fragmentomics [63,64,65]	Mixed tumor- and host-derived fragments reflecting chromatin organization, nucleosome positioning, fragment size, end motifs, and cleavage patterns	Fragmentation profiles may be affected by aging, inflammation, immune-cell turnover, tissue injury, exercise, organ dysfunction, and treatment-induced cell death	Genome-wide information without requiring predefined mutations; potential compatibility with low-input and multimodal assays	Limited tumor specificity; sensitivity to sample handling; dependence on sequencing depth, bioinformatic processing, and population-specific model performance	Early detection, cancer-signal classification, tissue-of-origin inference, tumor-fraction estimation, and disease monitoring
c-ncRNAs and EV-associated RNA cargo [20,66,67]	Tumor cells, immune cells, platelets, erythrocytes, endothelial cells, liver, adipose tissue, skeletal muscle, and other tissues; dynamic transcriptional and intercellular-signaling states	Strongly influenced by age, inflammation, diet, metabolic state, physical activity, medications, microbiome-related signals, tissue injury, and treatment exposure	Sensitivity to rapid biological and treatment-associated changes; potential information on immune, stromal, and metabolic adaptation	Hemolysis, platelet contamination, varied cellular and carrier origins, EV-isolation variability, inconsistent normalization, limited standardization, and tumor specificity	Prognosis, treatment-response monitoring, resistance assessment, immune and metabolic stratification, and exploratory early detection
Blood-cell DNA methylation clocks [9,10,68,69]	Predominantly leukocyte DNA; systemic biological aging, immune aging, and age-related physiological dysregulation	Influenced by chronological age, smoking, inflammation, blood-cell composition, adiposity, metabolic disease, nutrition, medication use, and treatment exposure	Established algorithms; compatibility with epidemiological and longitudinal survivorship studies	Dependence on clock selection and cell composition; population-specific performance; residual confounding; uncertain individual-level cancer prediction; not tumor-specific	Population-level risk research, survivorship studies, host-vulnerability assessment, and evaluation of treatment-associated aging

Abbreviations: cfDNA, cell-free DNA; c-ncRNAs, circulating non-coding RNAs; ctDNA, circulating tumor DNA; EVs, extracellular vesicles; MRD, molecular residual disease.

**Table 2:** Representative nutritional and microbiome-derived modifiers of epigenetic regulation across the aging–cancer continuum.

Nutrient or Modifier [Ref]	Principal Metabolic or Epigenetic Pathway	Potential Relevance to the Aging–Cancer Continuum	Current Evidence Level and Principal Limitations
Folate, vitamins B6 and B12, choline, betaine, methionine, serine, and glycine [142,143,144]	One-carbon metabolism, SAM: SAH balance, nucleotide synthesis, and redox regulation	May influence methylation capacity, genome stability, replication fidelity, biological aging, and tumor metabolism	Strong biochemical rationale; human outcomes are heterogeneous and depend on deficiency versus excess, genotype, tissue, dose, timing, and disease stage
Sulforaphane [145,146,147]	Inhibition of selected zinc-dependent histone deacetylases (HDACs) and activation of nuclear factor erythroid 2-related factor 2 (NRF2)-associated pathways	Experimental modulation of oxidative stress, inflammation, cell-cycle regulation, and chromatin acetylation	Predominantly preclinical evidence; limited short-term human biomarker data; exposure varies with food preparation, formulation, dose, and microbiome activity
Epigallocatechin-3-gallate (EGCG) [148,149,150]	DNMT inhibition and modulation of oxidative-stress and inflammatory pathways	Experimental re-expression of selected methylation-silenced genes and modulation of stress-related signaling	Mostly preclinical; low oral bioavailability, extensive metabolism, and non-specific effects limit clinical translation
Curcumin [151,152,153]	p300/CBP acetyltransferase-associated pathways, inflammatory transcription, and ncRNA regulation	Experimental effects on inflammation, epithelial–mesenchymal plasticity, cell survival, and ncRNA expression	Predominantly preclinical; pleiotropic activity, rapid metabolism, and low systemic bioavailability complicate interpretation
Resveratrol [154,155,156,157]	Sirtuin 1-associated pathways, AMP-activated protein kinase (AMPK), mitochondrial function, and redox signaling	Potential modulation of cellular stress responses, inflammation, metabolism, and mitochondrial function	Mixed mechanistic evidence; direct sirtuin activation is assay- and substrate-dependent; systemic exposure is limited
Butyrate and propionate [158,159,160,161]	Inhibition of selected HDACs, G-protein-coupled receptor signaling, and immune-metabolic regulation	Local modulation of intestinal epithelial function, inflammation, immune responses, and colorectal cancer biology	Strong mechanistic evidence for local effects; systemic concentrations are much lower than colonic exposure and vary with microbiome composition and substrate availability

Abbreviations: AMPK, AMP-activated protein kinase; DNMTs, DNA methyltransferases; EGCG, epigallocatechin-3-gallate; HDACs, histone deacetylases; NRF2, nuclear factor erythroid 2-related factor 2; SAH, S-adenosylhomocysteine; SAM, S-adenosylmethionine.

**Table 3:** Candidate biomarker and intervention strategies for monitoring or targeting epigenetic plasticity and therapeutic resistance.

Strategy	Principal Target or Rationale	Proposed Role	Current Evidence Level	Major Limitations and Priorities
Serial mutation-based ctDNA analysis [212,213,214]	Tumor-specific sequence variants, tumor burden, and clonal evolution	Early response assessment, MRD detection, recurrence surveillance, and resistance monitoring	Clinical validity demonstrated in several tumor-specific settings; clinical utility established in selected contexts but remains context-dependent	Requires trackable variants; limited sensitivity with low tumor shedding; interference from clonal hematopoiesis; treatment-changing thresholds and sampling schedules require prospective validation
cfDNA methylation and fragmentomics [58,215,216]	Cancer-associated methylation, tissue-of-origin signals, nucleosome organization, fragment size, end motifs, and genome-wide fragmentation patterns	Tumor-fraction estimation, treatment-response monitoring, molecular classification, and tissue-agnostic surveillance	Growing observational and clinical-validation evidence; limited evidence that biomarker-guided treatment decisions improve outcomes	Assay heterogeneity, low tumor fraction, pre-analytical sensitivity, sequencing-depth requirements, computational dependence, and limited prospective decision trials
c-ncRNA and EV profiling [217,218,219]	Dynamic transcriptional states, intercellular communication, immune adaptation, and non-genetic resistance mechanisms	Candidate prediction or monitoring of treatment response, epithelial–mesenchymal plasticity, immune adaptation, and chemoresistance	Predominantly preclinical and observational; limited clinical standardization	Hemolysis, platelet contamination, cellular and carrier heterogeneity, EV-isolation variability, inconsistent normalization, and limited tumor specificity
DNMTis [220,221,222]	DNA methylation maintenance, gene silencing, and broader nucleic-acid effects	Differentiation, re-expression of selected genes, immune modulation, chromatin priming, and combination therapy	Established clinical activity in selected hematological malignancies; investigational in many solid-tumor combinations	Myelosuppression, dose- and schedule-dependent effects, limited tumor specificity, adaptive resistance, and incomplete biomarker-based patient selection
HDACis [222,223,224]	Acetylation of histone and non-histone proteins	Modulation of transcription, differentiation, apoptosis, DNA repair, protein stability, and immune signaling	Established clinical activity in selected hematological malignancies; generally limited monotherapy activity in solid tumors	Pleiotropic effects, dose-limiting toxicity, incomplete target specificity, pharmacodynamic assessment, and limited identification of responsive subgroups
Fasting-mimicking interventions [203,204,225]	Glucose, insulin, IGF-1, nutrient-sensing pathways, systemic metabolism, and immune function	Investigational metabolic and immune modulation during anticancer treatment	Early-phase feasibility and biological-effect studies; definitive effects on progression-free or overall survival are not established	Nutritional risk, adherence, heterogeneous regimens, patient selection, treatment-specific safety, and absence of validated efficacy endpoints
Methionine restriction [206,226,227]	Methionine dependence, SAM metabolism, nucleotide synthesis, redox regulation, and protein synthesis	Investigational sensitization to radiotherapy or systemic anticancer treatment	Strong mechanistic and preclinical rationale; early phase I evidence of feasibility; no established clinical efficacy	Risk of inadequate protein and energy intake, uncertain target population, heterogeneous tumor dependence, adherence, and limited safety and efficacy data
Diet-derived bioactives and microbiome- derived metabolites [228,229,230]	DNMTs, HDACs, HATs, sirtuin-associated pathways, inflammatory signaling, metabolism, and ncRNA networks	Experimental modulation of chromatin, inflammation, metabolism, and treatment-associated signaling pathways	Predominantly mechanistic and preclinical, with limited short-term human biomarker evidence for selected compounds	Low or variable bioavailability, extensive metabolism, pleiotropic effects, supplement–drug interactions, variable microbiome responses, and limited standardized clinical trials
Integrated epigenetic–metabolic and mechanism-based combinations [177,231,232,233]	Concurrent targeting of chromatin plasticity, metabolic dependencies, immune adaptation, and DTP-associated vulnerabilities	Investigational disruption of adaptive persister states and targeting of adaptive persister-associated vulnerabilities and delay or overcoming of stable therapeutic resistance	Predominantly preclinical, with selected epigenetic–immune and metabolic combinations evaluated in early-phase trials	Overlapping tumor and host toxicity, adaptive pathway switching, treatment sequencing, therapeutic-window definition, biomarker selection, and complex trial design

Abbreviations: cfDNA, cell-free DNA; c-ncRNAs, circulating non-coding RNAs; ctDNA, circulating tumor DNA; DNMTis, DNA methyltransferase inhibitors; DNMTs, DNA methyltransferases; DTP, drug-tolerant persister; EVs, extracellular vesicles; HATs, histone acetyltransferases; HDACis, histone deacetylase inhibitors; HDACs, histone deacetylases; IGF-1, insulin-like growth factor 1; MRD, molecular residual disease; ncRNA, non-coding RNA; SAM, S-adenosylmethionine.

**Table 4:** Emerging technologies for resolving epigenetic aging and cancer-associated biomarkers.

Technology [Ref]	Principal Output	Main Contribution to the Aging–Cancer Continuum	Major Limitations	Current Translational Status
scATAC-seq and single-nucleus ATAC-seq [281,282,283]	Cell-specific chromatin accessibility, regulatory-element activity, and transcription-factor-associated programs	Resolves cellular heterogeneity, rare adaptive states, age-associated regulatory changes, and treatment-related chromatin plasticity that may be obscured in bulk analyses	Sparse data, batch effects, dissociation or nuclei-isolation bias, variable cell recovery, limited spatial information, and often small patient cohorts	Widely used for mechanistic research and biomarker discovery; limited routine clinical application
Single-cell DNA methylation Sequencing [260,284]	Cell-specific methylation profiles and lineage-associated epigenetic states	Directly assesses methylation heterogeneity and can identify rare cell populations, divergent aging trajectories, or tumor-associated epigenetic states	Low genomic coverage per cell, high cost, computational complexity, technical variability, and limited cohort sizes	Primarily research-stage, with emerging applications in lineage and cell-state analysis
Joint single-cell multi-omic profiling [285,286,287]	Paired or integrated chromatin-accessibility, transcriptional, protein, and/or methylation information from the same cell	Connects regulatory state with gene expression and phenotype, improving characterization of DTP, stem-like, immune, stromal, and senescent populations	Increased cost, missing data across modalities, difficult integration, lower throughput, and limited standardization	Rapidly developing; used mainly in discovery and mechanistic studies
Spatial epigenomics and transcriptomics [288,289,290]	Molecular states mapped within preserved tissue architecture	Retains anatomical and microenvironmental context and can identify resistant, senescent, hypoxic, invasive, or immune-excluded niches	Variable spatial resolution, limited tissue coverage, processing constraints, high cost, and evolving analytical standards	Emerging research technology with increasing relevance to tumor–microenvironment studies
Native long-read sequencing, including ONT and PacBio HiFi [291,292,293,294]	Native DNA sequence, modification signals, structural variants, haplotypes, and long-range genomic context	Enables conversion-free modification analysis, allele-specific methylation, haplotype phasing, and improved resolution of repetitive or structurally complex regions	DNA-input requirements, sequencing cost and throughput, platform-dependent modification calling, limited reference standards, and persistent low-tumor-fraction challenges in cfDNA	Rapidly developing for research and diagnostic development; not routine for most liquid-biopsy applications
Interpretable multimodal computational integration [295,296,297,298]	Integrated biomarker scores, tissue- or cell-of-origin estimates, tumor classification, biological-aging measures, and treatment-response or resistance predictions	Integrates tumor-derived biomarkers with host aging, nutritional, metabolic, microbial, imaging, and clinical information	High dimensionality, missing data, overfitting, information leakage, batch confounding, limited interpretability, calibration challenges, and need for independent cohorts	Increasingly used in biomarker development; clinical implementation remains limited and requires prospective evaluation

**Table 5:** Major translational sources of bias and recommended mitigation strategies in nutritional epigenetic biomarker research.

Challenge [Ref.]	Potential Consequence	Recommended Mitigation
Blood collection and processing variability [115,299]	Delayed processing, leukocyte lysis, residual cells, and inconsistent centrifugation may increase background genomic DNA and alter cfDNA concentration, fragmentomic profiles, or the relative ctDNA fraction	Prespecify collection tubes, maximum processing intervals, centrifugation conditions, plasma volume, storage procedures, and freeze–thaw limits; use validated stabilizing tubes when immediate processing is not feasible
Hemolysis and platelet contamination [218,219]	Release of erythrocyte- and platelet-derived RNAs and EVs may distort c-ncRNA profiles and create apparent disease-associated differences	Assess hemolysis using predefined visual, spectrophotometric, or molecular criteria; standardize platelet depletion and report residual platelet contamination
Assay-specific analytical bias [115,218,219]	DNA loss, incomplete or uneven methylation conversion, amplification artifacts, variable CpG coverage, inconsistent EV isolation, or unsuitable RNA normalization may reduce reproducibility	Use validated low-input workflows, reference materials, process controls, spike-ins where appropriate, prespecified QC thresholds, and transparent reporting of sensitivity, precision, extraction recovery, and failure rates
Dietary exposure misclassification and temporal mismatch [300,301]	Recall error, inaccurate portion estimation, under-reporting, and single-time-point measurements may distort diet–biomarker associations or compare short-term exposure with long-term epigenetic outcomes	Combine repeated validated dietary assessments with objective biomarkers, metabolomics, supplement records, and sampling schedules matched to the expected biological timescale
Metabolic and microbiome heterogeneity [301]	Similar reported diets may generate different concentrations of bioactive metabolites because of variation in microbial composition, intestinal physiology, organ function, medications, and host metabolism	Characterize microbiome composition, metabolomic profiles, medication use, gastrointestinal and metabolic factors, body composition, and relevant host phenotypes
Cell-composition and host-state confounding [302,303]	Changes in blood-cell proportions, inflammation, tissue injury, or treatment exposure may be misinterpreted as tumor-specific or nutrition-associated epigenetic alterations	Incorporate measured blood counts, validated cell-type deconvolution, purified-cell analyses where feasible, and adjustment for inflammatory, hematological, and treatment-related variables
Confounding and reverse causality [304]	Lifestyle, socioeconomic, metabolic, and clinical factors may influence both exposure and outcome, while preclinical cancer may alter diet, body composition, inflammation, metabolism, and circulating biomarkers	Use prospective sampling, serial measurements, lagged analyses, exclusion of early events, negative controls, directed causal models, and triangulation across complementary study designs
Invalid or weak causal instruments [304,305]	Horizontal pleiotropy, linkage disequilibrium, weak instruments, population stratification, and tissue mismatch may bias Mendelian-randomization estimates	Assess instrument strength and assumptions; apply pleiotropy-robust sensitivity analyses, bidirectional approaches, multiple independent instruments, and formal colocalization where appropriate
Overfitting, information leakage, and poor calibration [306,307]	Model performance may be inflated during development and decline substantially in independent patients or clinical settings	Confine preprocessing and feature selection to training data; use patient-level separation, nested cross-validation, locked algorithms, calibration assessment, and independent external validation
Population underrepresentation [306,308]	Biomarkers and algorithms may show reduced accuracy, transportability, or clinical benefit in underrepresented demographic or clinical groups	Recruit diverse and clinically representative populations; report subgroup discrimination, calibration, and utility; update or recalibrate models when transported to new settings
Unvalidated intermediate or surrogate endpoints [309,310]	Improvement in a biomarker, including an epigenetic-aging measure, may not correspond to reduced recurrence, prolonged survival, lower toxicity, or improved quality of life	Treat candidate biomarkers as exploratory or mechanistic outcomes until validated; prospectively link biomarker changes to patient-relevant endpoints and state the limitations of surrogate use
Incomplete reporting and insufficient data harmonization [218,305,306]	Inadequate reporting of protocols, metadata, processing steps, code, and model versions limits replication, comparison, and meta-analysis	Preregister confirmatory analyses; follow assay- and design-specific reporting guidance; use harmonized metadata, standardized terminology, interoperable formats, and version-controlled code

Abbreviations: cfDNA, cell-free DNA; c-ncRNAs, circulating non-coding RNAs; ctDNA, circulating tumor DNA; EVs, extracellular vesicles.

## Data Availability

No new data were created or analyzed in this study. Data sharing is not applicable to this article.
